# Sperm storage reduces the strength of the mate‐finding Allee effect

**DOI:** 10.1002/ece3.6019

**Published:** 2020-02-07

**Authors:** María V. Jiménez‐Franco, Andrés Giménez, Roberto C. Rodríguez‐Caro, Ana Sanz‐Aguilar, Francisco Botella, José D. Anadón, Thorsten Wiegand, Eva Graciá

**Affiliations:** ^1^ Ecology Area Deparment of Applied Biology Miguel Hernández University ‐ Av. de la Universidad. Torreblanca Elche Spain; ^2^ Department of Ecological Modeling UFZ–Helmholtz Centre for Environmental Research Leipzig Germany; ^3^ Departamento de Ecología Universidad de Alicante Alicante Spain; ^4^ Animal Demography and Ecology Unit IMEDEA (CSIC‐UIB) Esporles Spain; ^5^ Applied Zoology and Conservation Group University of Balearic Islands Palma Spain; ^6^ Department of Biology Queens College, City University of New York Flushing NY USA; ^7^ The Graduate Center, Biology Program City University of New York New York NY USA; ^8^ Área de Ecología Departamento de Ciencias Agrarias y el Medio Natural Universidad de Zaragoza Huesca Spain; ^9^ German Centre for Integrative Biodiversity Research (iDiv) Halle‐Jena‐Leipzig Leipzig Germany

**Keywords:** human‐altered landscape, individual‐based modeling, limited movement ability, low‐density population, population extinction, population growth rate, reproductive rate, *Testudo graeca*

## Abstract

Mate searching is a key component of sexual reproduction that can have important implications for population viability, especially for the mate‐finding Allee effect. Interannual sperm storage by females may be an adaptation that potentially attenuates mate limitation, but the demographic consequences of this functional trait have not been studied. Our goal is to assess the effect of female sperm storage durability on the strength of the mate‐finding Allee effect and the viability of populations subject to low population density and habitat alteration. We used an individual‐based simulation model that incorporates realistic representations of the demographic and spatial processes of our model species, the spur‐thighed tortoise (*Testudo graeca*). This allowed for a detailed assessment of reproductive rates, population growth rates, and extinction probabilities. We also studied the relationship between the number of reproductive males and the reproductive rates for scenarios combining different levels of sperm storage durability, initial population density, and landscape alteration. Our results showed that simulated populations parameterized with the field‐observed demographic rates collapsed for short sperm storage durability, but were viable for a durability of one year or longer. In contrast, the simulated populations with a low initial density were only viable in human‐altered landscapes for sperm storage durability of 4 years. We find that sperm storage is an effective mechanism that can reduce the strength of the mate‐finding Allee effect and contribute to the persistence of low‐density populations. Our study highlights the key role of sperm storage in the dynamics of species with limited movement ability to facilitate reproduction in patchy landscapes or during population expansion. This study represents the first quantification of the effect of sperm storage durability on population dynamics in different landscapes and population scenarios.

## Graphical abstract

We find that sperm storage is an effective mechanism that can reduce the strength of the mate‐finding Allee effect and contribute to the persistence of low‐density populations, especially in altered landscapes. Our study represents the first quantification of the effect of sperm storage durability on population dynamics in different landscapes and population scenarios.

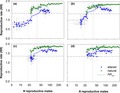

## 1 INTRODUCTION

Mate searching is one of the most important reproductive processes for animal species with sexual reproduction (Berec & Boukal, 2004). However, individuals may fail to find mates when population density is low (Berec, Kramer, Bernhauerová, & Drake, 2018; Gascoigne, Berec, Gregory, & Courchamp, 2009). This effect might be amplified in patchy landscapes (Walter, Firebaugh, Tobin, & Haynes, 2016), especially for species with limited movement ability (Anthonysamy, Dreslik, Douglas, Marioni, & Phillips, 2014). Mate limitation has major consequences for reproductive fitness and can cause a mate‐finding Allee effect (Berec, Boukal, & Berec, 2001; Gascoigne et al., 2009). More generally, an Allee effect can be defined for small population densities as “a density‐dependent phenomenon in which population growth or individual components of fitness increase as population density increases” (Kramer, Berec, & Drake, 2018). Nevertheless, there are different adaptations in sexual reproduction that facilitate mate finding, such as the ability to maintain long‐term pair bonds, signaling (e.g., calls, pheromones), breeding site fidelity, or induced ovulation (Gascoigne et al., 2009). Some reproductive systems, like density‐dependent sex determination, sperm storage, parthenogenesis, or hermaphroditism, represent solutions that allow reproduction with low mating frequency or even without mating (Gascoigne et al., 2009; Orr & Zuk, 2012).

Sperm storage is “the maintenance of sperm inside a female's reproductive tract for an extended period of time” (Orr & Zuk, 2012). It plays an important role in species reproduction in which male and female cycles do not coincide (Roques, Díaz‐Paniagua, Portheault, Pérez‐Santigosa, & Hidalgo‐Vila, 2006) as it allows fertilization without the need to mate immediately prior to reproduction (Pearse, Janzen, & Avise, 2002; Roques, Díaz‐Paniagua, & Andreu, 2004). This reproductive adaptation is found in a wide range of animals: insects, arachnids, fishes, reptiles, birds, or mammals (Aral & Sahin, 2015; Baer, Collins, Maalaps, & den Boer, 2016; Fromhage, Jennions, & Kokko, 2016). The viability of stored sperm varies from hours to days (in mammals, including humans), and even for years (Holt & Fazeli, 2016), and is especially long‐lasting in reptiles (Cutuli, Cannicci, Vannini, & Fratini, 2013). While the location in female specialized structures, molecular basis, and duration of sperm storage have been well‐studied in different taxa (Orr & Brennan, 2015), very little attention has been paid to the ecological role of sperm storage in population dynamics (López‐Sepulcre, Gordon, Paterson, Bentzen, & Reznick, 2013).

The general objective of this study was to assess the effect of female sperm storage durability on the strength of the mate‐finding Allee effect and the viability of populations subject to low population density and habitat alteration. To address our general objective, we used the spatially explicit individual‐based model STEPLAND (Graciá et al., 2020), which synthesizes data from almost two decades of intense field studies into the spatial and demographic processes of our model species, the spur‐thighed tortoise (*Testudo graeca*), in southeast Spain. This species is ideally suited to address our general objective because it is a long‐lived species with late sexual maturity and limited movement ability. It is also known that *T. graeca* females can store viable sperm from at least one year (Roques et al., 2004) to several years (own data). Because of these characteristics, we suspect that mate‐finding Allee effects may be present in *T. graeca* populations (Graciá et al., 2013). The movement ecology and demography of *T. graeca* in both natural and human‐altered landscapes is relatively well‐known (e.g., Anadón, Giménez, Martínez, Palazón, & Esteve, 2007; Anadón, Wiegand, & Giménez, 2012; Pérez, Giménez, Anadón, Martínez, & Esteve, 2002; Rodríguez‐Caro, Graciá, Anadón, & Giménez, 2013; Sanz‐Aguilar et al., 2011).

The simulation model STEPLAND integrates many pieces of information provided by our data on the spatially explicit movement of individual tortoises and of demographic processes, including the sperm storage mechanism, into a common logical framework. This allows us to explore the long‐term consequences of such information for different scenarios of real‐world landscape structure and sperm storage durability. Such a long‐term assessment would be impossible within the frame that usual empirical studies take. More specifically, we used this simulation model to investigate the effects of different sperm storage durabilities (ranging from the same breeding season to 4 years) on reproductive rates (the mean number of yearlings produced by reproductive females per year), population growth rates, extinction probabilities, and the mate‐finding Allee effect (i.e., the relationship between the number of reproductive males and the reproductive rate; Levitan, 2002). These effects were evaluated in four scenarios that combined natural and human‐altered landscapes, plus two initial population densities. We hypothesized that: 1) sperm storage durability is an essential reproductive trait to avoid some of the negative consequences of mate‐finding Allee effects (e.g., extinction); 2) longer sperm storage duration should increase the population viability, especially for populations with low density and/or located in human‐altered landscapes.

## 2 MATERIALS AND METHODS

### 2.1 Study system: the spur‐thighed tortoise in southeast Spain

The spur‐thighed tortoise *T. graeca* is a medium‐sized tortoise with an annual reproductive period. Courtships are observed through their breeding season in spring, in which males are very active to search for females (Díaz‐Paniagua, Keller, & Andreu, 1995). Females are able to store sperm from either single mating or multiple mating, at least within a breeding season, to allow clutching without mating immediately prior to reproduction (Roques et al., 2004). Although the longevity of stored sperm in *T. graeca* has not been formally documented, according to personal communications from breeders at wildlife rehabilitation centers, it could be viable in the oviducts of females for 3–4 years, as found for Hermann's tortoise (*T. hermanni*; Cutuli et al., 2013). Females harboring viable sperm lay up to three clutches in spring (Díaz‐Paniagua, Keller, & Andreu, 1996), with clutch sizes varying between 1 and 7 eggs (the authors, unpublished data).

The main *T. graeca* population in Western Europe is located in southeast Spain, where it inhabits heterogeneous landscapes consisting of a shrubland matrix interspersed with other land uses, for example, mainly irrigated and nonirrigated crops (Anadón et al., 2006). The main threats to this population are habitat loss and fragmentation, both caused by anthropogenic activities (e.g., irrigated lands, greenhouses, and tourism) (Anadón, Giménez, Ballestar, & Pérez, 2009). Given *T. graeca's* limited movement ability, such habitat alterations can cause population fragmentation at the landscape levels (Anadón et al., 2012).

### 2.2 Deterministic demographic model

The population dynamics of *T. graeca* is influenced by a “deterministic skeleton” (driven by the age‐dependent demographic rates), demographic stochasticity, and spatial effects (e.g., a mate‐finding Allee effect). To represent the deterministic skeleton, we developed a Leslie projection matrix model (Caswell, 2001) based on the mean values of our demographic parameters (Appendix S1), assuming that all reproductive females mated (i.e., no spatial effects or demographic stochasticity). The *popbio* package (Stubben & Milligan, 2007) in the statistical program R version 3.5.1 (R Core Team, 2016; R scripts are shown in Appendix S2) was used for the analyses of the Leslie matrix model.

We used the stable age distribution predicted by the Leslie model to determine the initial population (i.e., the number, ages, and sex of individuals) for the STEPLAND simulations (see the calculation details in Appendix S2), and assuming a sex ratio of 0.5 since the majority of the *T. graeca* populations show balanced sex ratios (Graciá et al., 2017).

We also used two population characteristics, the deterministic population growth rate *λ_det_* and the deterministic reproductive rate *RR_det_*, as predicted by the Leslie model, to assess the effects of demographic stochasticity and spatial effects in the STEPLAND simulations. The reproductive rate *RR* (i.e., the mean number of yearlings produced by reproductive females per year) was estimated as *RR* = *NC*×CS × *ES*×MP*,* with *NC* (mean number of clutches), *CS* (mean number of eggs per clutch), *ES* (mean hatching success × survival rate of individuals below 1 year), and *MP* (proportion of females mating) (Appendix S2). For estimation of *RR_det,_* we assumed *MP* = 1, but in the STEPLAND simulations spatial effects may lead to mating failure (i.e., *MP* < 1) and reduce the values of the simulated *RR* and *λ* in simulation scenarios with low population densities. The purpose of the model simulations was to quantify these effects.

Finally, we used the Leslie transition matrix model to determine the “critical” reproductive rate *RR_crit_* that corresponded to a critical growth rate of 1 (*λ_crit_* = 1). For reproductive rates below *RR_crit_*, the population is likely to become deterministically extinct.

### 2.3 STEPLAND: a spatially explicit individual‐based model to simulate spur‐thighed populations’ viability

**Figure 1:**
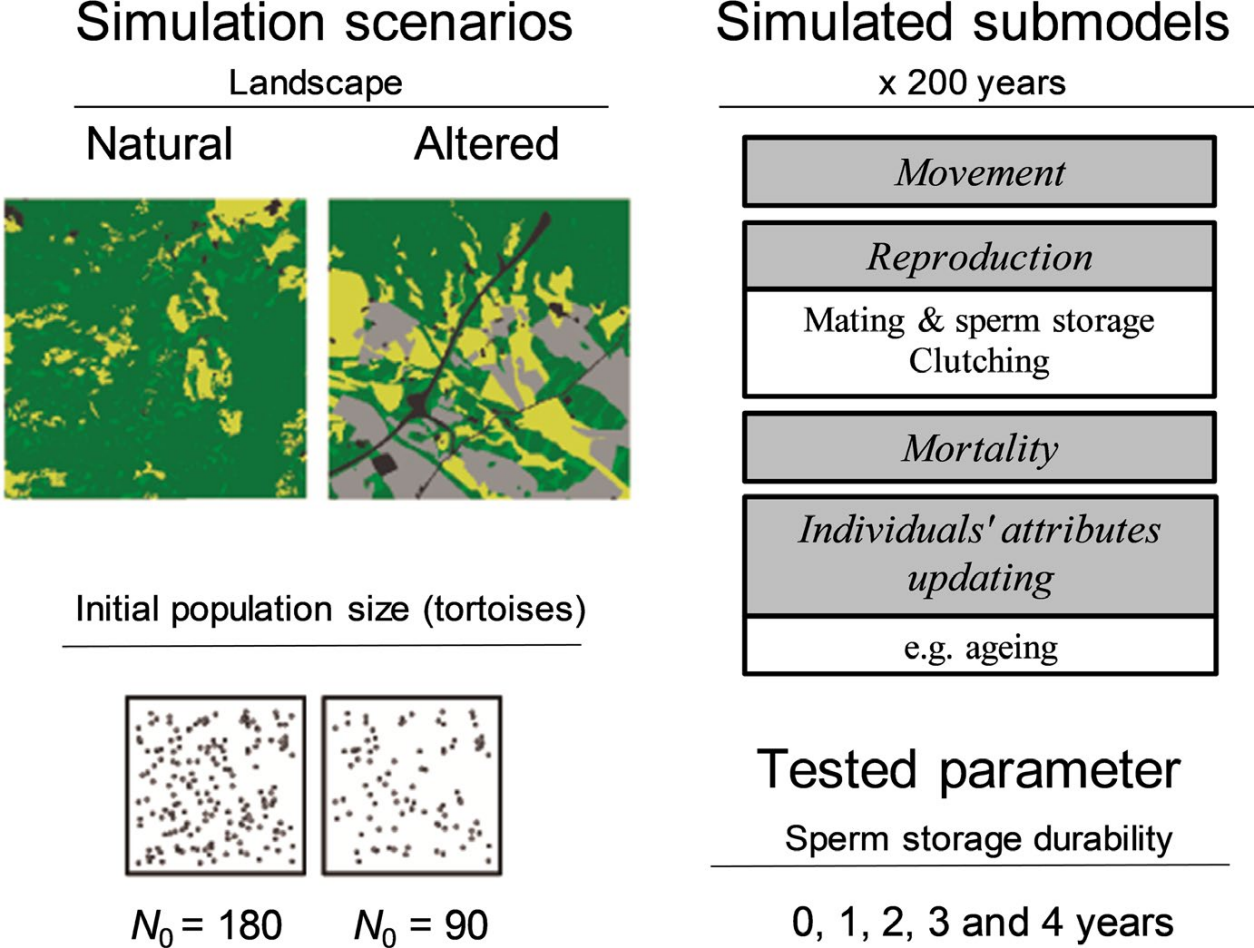
General overview of the individual‐based model STEPLAND that focused on studying the effects of different sperm storage durabilities in a species with limited movement ability: *Testudo graeca*. We simulated the population dynamics in two different real landscapes: a natural landscape and an altered landscape. Habitat categories include: nonpermeable infrastructures (black), intensive lands use (gray), traditional agriculture lands (yellow), natural flat areas (light green), and natural areas on slopes (dark green). The simulations varied the initial population size (*N*_0_ = 90 and 180) and sperm storage durability (from 0 to 4 years) for a 200‐year simulation period

The STEPLAND model was developed to integrate demographic processes (i.e., reproduction, mortality, and aging; Graciá et al., 2020) with a previously developed individual‐based model of tortoise movement (Anadón et al., 2012). For a model description, we followed the Overview, Design concepts and Details protocol (ODD) proposed by Grimm et al. (2010). We summarize the model in the paragraphs below. A full ODD version is provided in Appendix S3. The model was implemented into Python 2.7 and its code, parameterization, and the main results files are available in the figshare repository (Jiménez‐Franco et al., 2020). Figure 1 provides a general overview of the model (with the movement and demographic submodel) and the simulation experiments, including the different scenarios of landscape, initial population size, and sperm storage durability.

Landscapes were composed of a grid of 10 m × 10 m cells. We used two typical 3 km × 3 km real landscapes from the Almenara Mountains in SE Spain that currently host *T. graeca* populations, based on a previous study of radiotracked individuals (Anadón et al., 2012) (Figure 1): “Galera” represents a natural landscape with very low intensive agricultural land uses or nonpermeable infrastructures (1%), and “Bas” represents a human‐altered landscape with a higher proportion of intensive agricultural land uses and nonpermeable infrastructures (26%). We considered two different initial population densities (0.2 and 0.1 tortoises/ha) to represent scenarios of medium and low population densities of *T. graeca* in natural Spanish populations (Anadón et al., 2009). The corresponding initial population sizes were 180 and 90 individuals in 3 km × 3 km landscapes for the scenarios of initial population density of 0.2 and 0.1 tortoises/ha, respectively. The different age classes of the initial population were based on the stable age distribution (Rodríguez‐Caro et al., 2019; see details in the deterministic demographic model section and Appendix S2), and tortoises were randomly distributed across suitable habitats in each landscape.

Tortoise movement in the model consisted in a sequence of steps to neighbor 10 m × 10 m cells, as described by Anadón et al. (2012), and according to radiotracking data. The stochastic rules that determined the number of these “cell‐to‐cell” steps were governed by probabilities PMOV and DMOV, which were directly taken from detailed radiotracking data. To this end, the active movement period of each day was divided into four intervals (defined by five daily radiotracking locations 2–3 hr apart), where PMOV decided if the individual moved within a given interval. If this was the case, DMOV decided how many cell‐to‐cell steps the individual moved. PMOV and DMOV depended on the month of the year and differed between males and females (see Appendix S3, Figure S4a,b). While PMOV and DMOV govern the number of cell‐to‐cell steps that an individual moved, the actual selection of one of the eight neighboring cells depended on three sets of weights that described autocorrelation in movement, home behavior, and habitat dependence (see Appendix S3, *Movement*). Weights were introduced in the model by the nine additional parameters determined in Anadón et al. (2012) (see Appendix S3 for further details). Because cells with highways and urban areas (nonpermeable infrastructures) received weights of zero, they represent barriers for movement. Intensive agriculture received low weights, which means that they can be crossed, but less frequently than natural areas or traditional agricultural areas.

The demographic parameters reflect the species’ biology (Appendix S1). Reproduction consists of three processes: mating, sperm storage, and clutching (Figure 1). Mating occurred once during the mating season (spring) when movement activity peaked (Appendix S3, Figure S4). Mating in *T. graeca* is a complex process, and the attraction mechanisms of reproductive individuals are far unknown. Given the lack of detailed information on mate finding, we implemented this process in a simple way by assuming that females will mate if during the peak mating season in spring (at the end of April) at least one reproductive male is located within a given encounter distance DIST. We selected a value of DIST = 500 m. This value was based on (a) a sensitivity analysis of Graciá et al. (2020) that evaluated the effect of DIST values between 100 m and 700 m on population dynamics and (b) mean maximal annual displacement distances of radiotracked *T. graeca* males and females (189–275 m and 148–271 m, respectively; Anadón et al., 2012). Therefore, the STEPLAND model assumed that mature females and males were able to mate if: (a) the distance between female and male was shorter than the parameter DIST value (= 500 m; i.e., “mating excursions” were not explicitly modeled); (b) females and males were not separated by barriers such as main roads, dense human infrastructures or intensive agriculture. The Galera landscape lacks internal barriers, but barriers (e.g., roads and intensive agriculture) exist in the Bas landscape (Figure S1 of Appendix S3).

Only adult individuals (males aged ≥7 years, females aged ≥10 years) reproduce (Rodríguez‐Caro et al., 2013; Sanz‐Aguilar et al., 2011). Sperm storage was modeled in a simple way: The females that find males reproduce within the next *n* years, without further mating being necessary (*n* = 0–4, depending on the scenario). For the scenarios with *n* = 0, sperm is only viable during the current breeding season, whereas the scenario with *n* = 4 allows females to reproduce for the next 4 years after mating. To be conservative, we assumed that sperm viability was 100% within the next *n* years, but 0% afterward. Regarding clutching, the model assumed that the adult females harboring viable sperm had the opportunity to lay between one to three clutches per year, as in real populations (Díaz‐Paniagua et al., 1996). The eggs were placed at the location of the female in spring and early summer when clutching occurs (from the end of April to the end of July), and the newborns emerge from this position. Annual survival rates are applied at the end of the year and vary among age classes, including newborns (representing hatching success and survivals of individuals below 1 year), immature individuals (aged 1–3), subadults (aged 4–6), and adults (aged ≥7). Appendix S1 provides the parameters values and an extended description of the criteria followed for their selection. Data output consists of CSV files that comprise the surviving tortoises of the population and their attributes (location in the landscape, gender and age) in steps of *T* = 10 years for the 200 simulated years (Jiménez‐Franco et al., 2020).

### 2.4 Design of the simulation experiments

We evaluated the effect of female sperm storage durability on three key demographic variables: the reproductive rate *RR*, the growth rate *λ,* and the probability of extinction *P*_ext_ over a 200‐year period. To this end, we repeated 64 replicates for each scenario, which were composed by two types of landscape and two initial population sizes (see the details above) for different sperm storage durability values (from 0 year, which represents sperm storage only during the current reproductive season, up to 4 years). The total number of independent model simulations was 1,280 (4 simulation scenarios × 5 values of sperm storage duration × 64 replicates).

### 2.5 Data analysis

Data were obtained from the simulated populations (CSV files with information about surviving tortoises) and were processed to obtain the population variables (*RR*, *λ,* and *P*_ext_) averaged over the 64 replicate trajectories per scenario for each year *t* (measured in steps of *T* = 10 years) by means of the matrix calculations in R version 3.5.1 (R Core Team, 2016). We calculated the extinction rate *P*_ext_ for each year *t* as the proportion of extinct replicates (i.e., having a population size *N_t_* = 0). The growth rate *λ_t_* was calculated for each interval (*t* – *T*, *t*) as:

(1) $$\lambda_{t}={\left(\frac{N_{t}}{N_{t-T}}\right)}^{\text{1}\;/T}$$

The reproductive rate *RR* at time step *t* was the mean number of offspring of reproductive females and calculated by dividing the number of individuals born in year *t* by the number of reproductive females (those aged 10 years of older) in year *t*. Finally, for all the simulated scenarios, we calculated the number of reproductive males (aged ≥7 years) for each year *t* and related them with *RR* (as a measure of population fitness) to show the Allee effect for a low‐density population (Levitan, 2002). Only the nonextinct trajectories at each time were used to calculate the number of reproductive males, *RR,* and *λ*.

## 3 RESULTS

The Leslie transition matrix model used to establish initial demographic structure predicted a deterministic growth rate of *λ_det_* = 1.008 and a reproductive rate *RR_det_* = 1.7. Moreover, a critical population growth rate *λ_crit_* = 1 resulted from a “critical” *RR_crit_* = 1.35 and a “critical” mating probability *MP_crit_* = 0.79.

**Figure 2:**
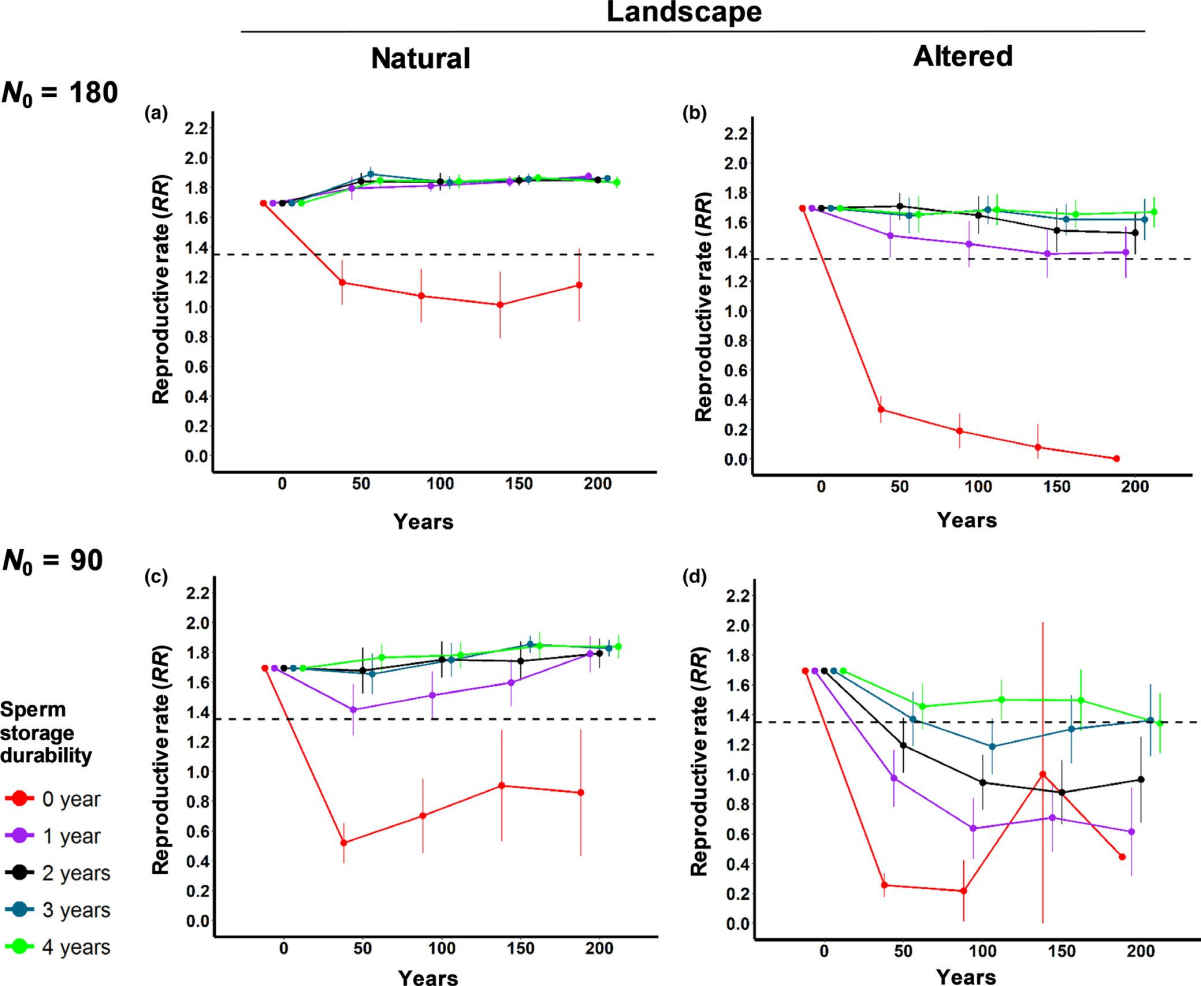
Mean reproductive rates (*RR*, mean number of offspring per reproductive female) of *T. graeca* for the different simulation scenarios for 200 years. The *RR* (mean ± confidence intervals) estimates were calculated only for the simulations that did not become extinct. The dashed black lines show the critical *RR_crit_* that would result from a critical growth rate of 1. Note that scenario d) does not show a confidence interval for the 0‐year sperm storage durability due to a small sample size (*n* = 5). The different sperm storage durability scenarios (0, 1, 2, 3, and 4 years) are indicated by different colors

**Figure 3:**
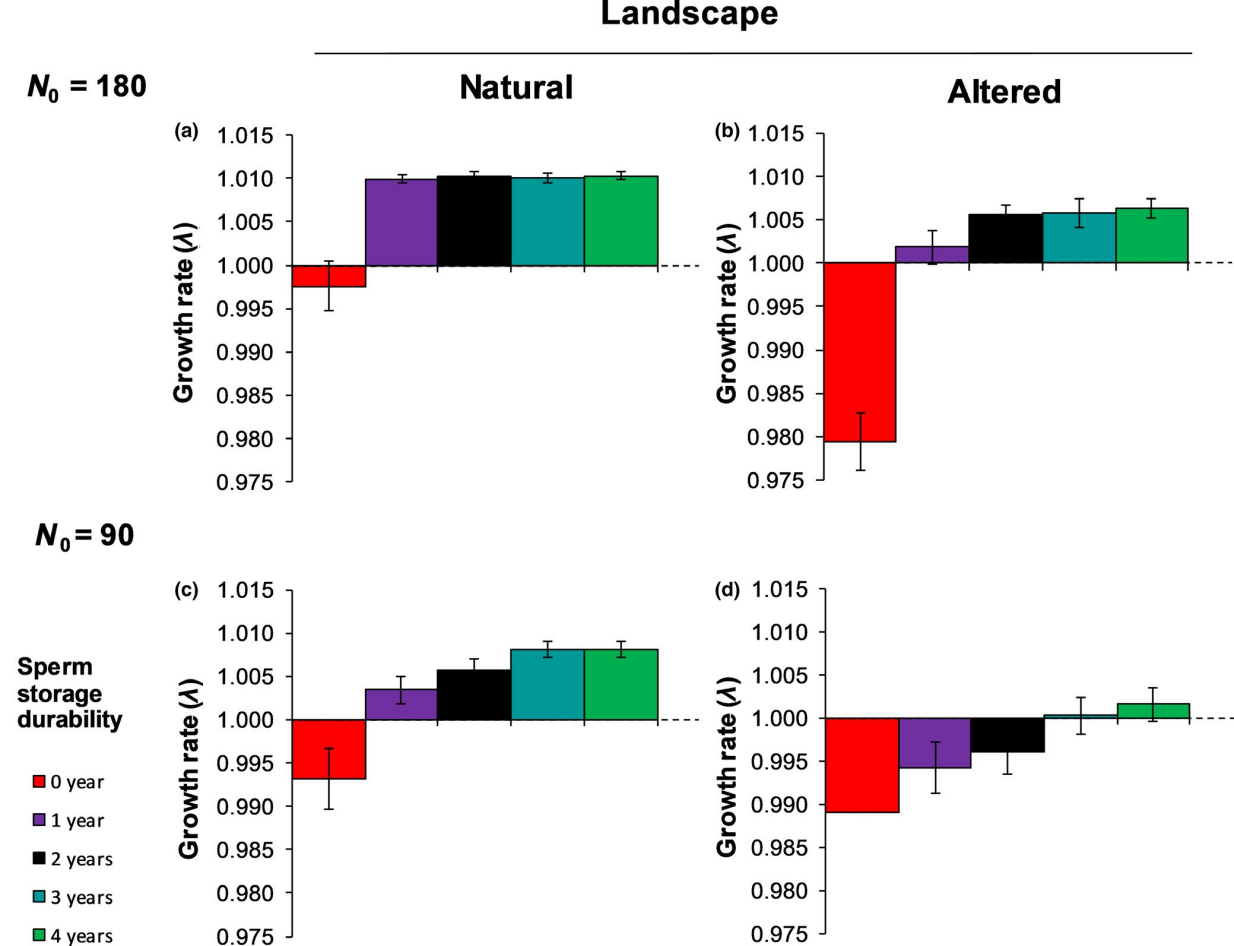
Population growth rate (λ_t_) of *T. graeca* (mean and confidence intervals) by year *t* = 200. The dotted black line shows the critical population estimates *λ_crit_*. Growth rates were calculated only for the simulated populations that did not become extinct. Note that scenario d) does not show any confidence interval for the 0‐year sperm storage durability due to a small sample size (*n* = 5). The different sperm storage durability scenarios (0, 1, 2, 3, and 4 years) are indicated by different colors

**Figure 4:**
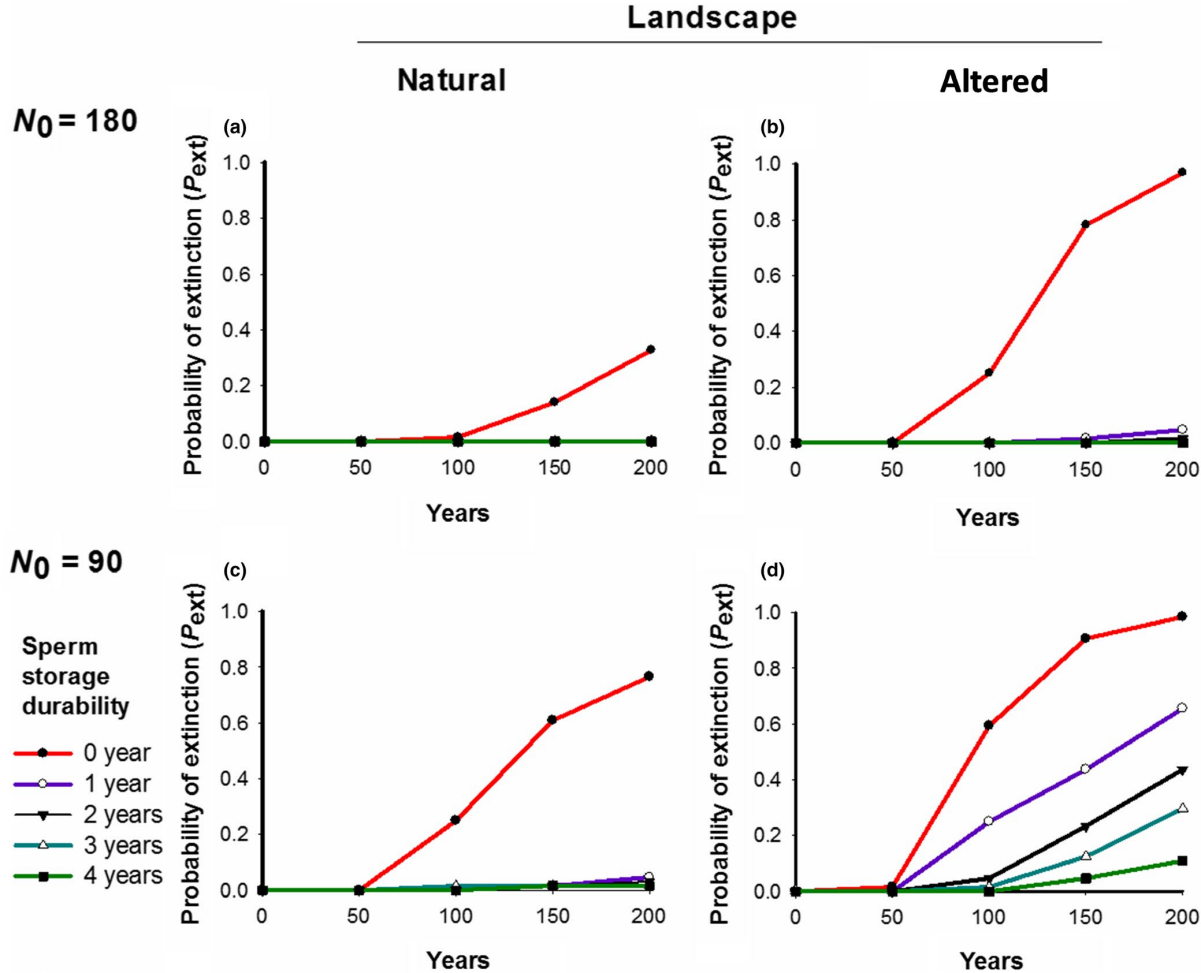
Probability of the extinction (*P*_ext_) of *T. graeca* for the different simulated scenarios for 200 years. The different sperm storage durability scenarios (0, 1, 2, 3, and 4 years) are indicated by different colors

Populations where sperm storage durability was limited to the current breeding season (0 years) showed *RR* values below the critical threshold of *RR_crit_* = 1.35 in all the scenarios (Figure 2), showed population growth rates lower than one (Figure 3) and a high risk of extinction (Figure 4). Except for the scenario with the altered landscape and a small initial population size (Figures 2d, 3d, and 4d), a sperm storage durability of one year resulted in reproductive rates *RR* above the critical threshold of 1.35 (Figure 2a‐c), population growth rates higher than one (Figure 3a‐c), and a risk of extinction below 5% after 200 years (Figure 4a‐c).

**Figure 5:**
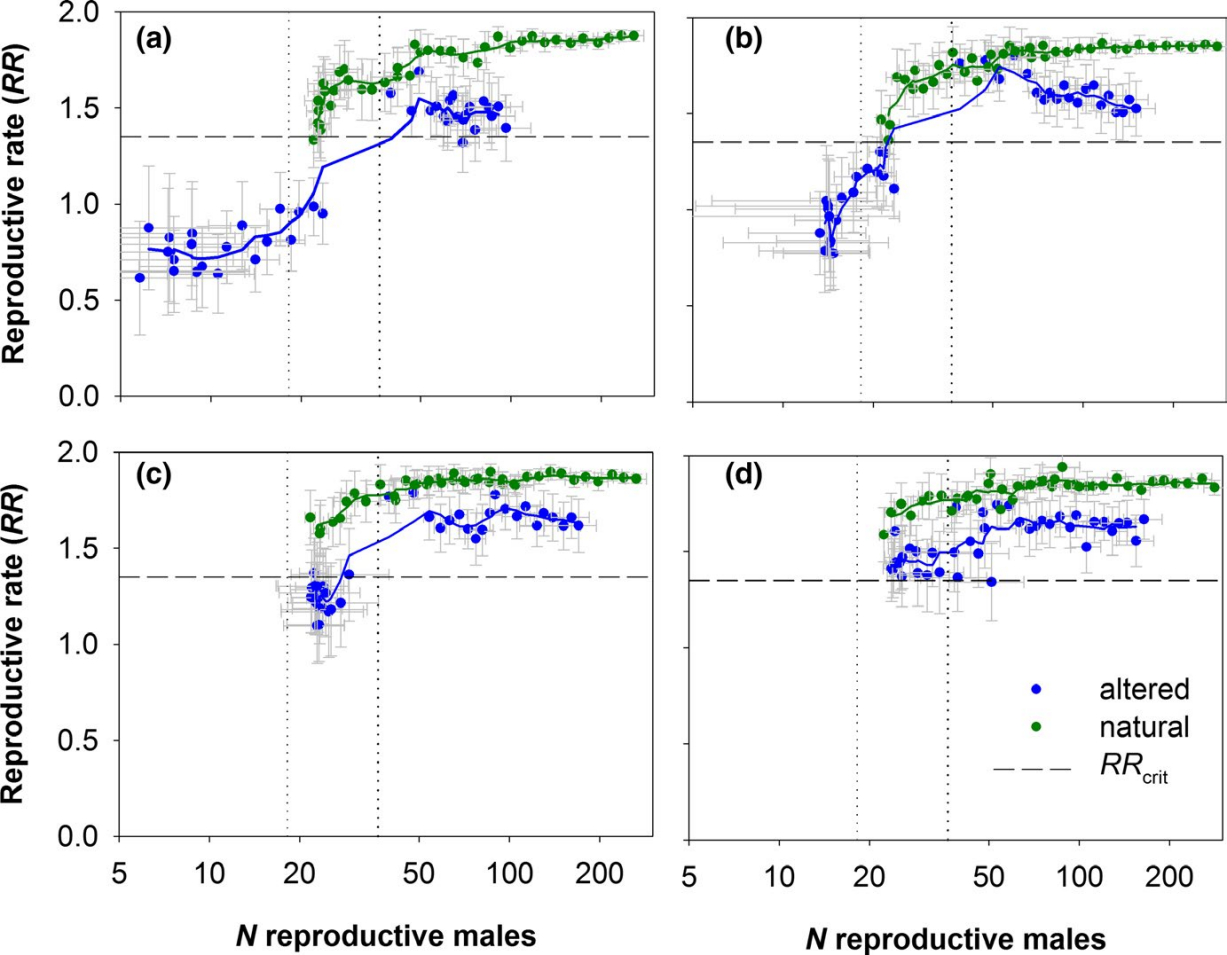
The relationship between the number of reproductive males and the *RR* (mean ± confidence intervals) of *T. graeca* for each sperm storage durability from 1 up to 4 years (a‐d, respectively). Dots represent the different simulated years, and colors denote the landscape scenarios (natural and altered) for the two initial population sizes (*N*_0_ = 90 and 180). The dashed horizontal line shows the critical population estimate *RR_crit_,* while the dotted vertical lines denote the initial number of reproductive males in the two initial population density scenarios (18 and 36)

Analyses of the demographic rates showed that the populations with initially 180 individuals exceeded the critical growth rate of *λ_crit_* = 1 and the critical reproductive rate *RR*_crit_ = 1.35 if sperm storage durability lasted at least one year (Figures 2a, 3a, respectively) and approximated the theoretical estimates of *λ_det_* = 1.008 and *RR_det_* = 1.7 expected for cases without demographic stochasticity and mate limitation. Thus, demographic stochasticity and spatial effects played a minor role for high‐density populations in natural landscapes. However, in all other scenarios, the population growth rates clearly increased when sperm storage durability was increasing (Figure 3b–d). Our results show a clear mate‐finding Allee effect: The reproductive rates *RR* were low when the number of males was low, but they increased as the number of males was increasing (Figure 5). Moreover, the Allee effect was stronger for scenarios in the altered landscape than for scenarios in the natural landscape (Figure 5; Table 1), and its strength increased with decreasing sperm storage durability (Figure 5; Table 1).

**Table 1:** Strength of the Allee effect in response to sperm storage durability. The mean value of the reproductive rate estimated for the two initial population sizes *RR*(*N*_0_ = 90) and *RR*(*N*_0_ = 180) over the 200 simulation years for each sperm storage durability. The difference *RR*(*N*_0_ = 180) – *RR*(*N*_0_ = 90) can be interpreted as an indicator of the strength of the mate‐finding Allee effect

Sperm storage durability (years)	*RR*(*N*_0_ = 90)	*RR*(*N*_0_ = 180)	Difference	Landscape
0	–	–	–	Altered*
1	0.793	1.484	0.691	Altered
2	1.022	1.619	0.597	Altered
3	1.243	1.664	0.421	Altered
4	1.455	1.654	0.198	Altered
0	0.780	1.091	0.311	Natural
1	1.569	1.824	0.255	Natural
2	1.683	1.837	0.153	Natural
3	1.771	1.859	0.088	Natural
4	1.783	1.848	0.065	Natural

* Almost all the populations became extinct.

## 4 DISCUSSION

Reproductive adaptations, such as sperm storage, could be essential traits for the persistence of species with mating difficulties due to low density and limited movement ability and would be a mechanism to reduce the strength of the mate‐finding Allee effect. Some studies have evaluated the biological consequences of mate‐finding limitation (Carver, Wolcott, Wolcott, & Hines, 2005; Fromhage et al., 2016; Gascoigne et al., 2009) and have even implemented individual‐based models to address this question (Berec & Boukal, 2004; Berec et al., 2001, 2018; Coates & Hovel, 2014; Engen, Lande, & Sæther, 2003; Gregory, Bradshaw, Brook, & Courchamp, 2010; Walter et al., 2016). However, to the best of our knowledge, this is the first study to assess the ecological role of sperm storage in relation to the mate‐finding Allee effect in a spatial context. By using an individual‐based model that combines our field data on demographic parameters and movement patterns with real landscapes, we assessed the effect of different sperm storage durabilities on the dynamics and the risk of extinction of *T. graeca* populations with different initial population sizes and that live in natural versus. altered landscapes.

Our results suggest that the mate‐finding Allee effect occurs in the study species *T. graeca*: The reproductive rates were positively correlated with the number of males, and the Allee effect was stronger in the simulations of low‐density scenarios than in the high‐density scenarios. This effect emerged due to the difficulty of mate finding at low densities (Stephens, Sutherland, & Freckleton, 1999), and such difficulties became particularly severe in the human‐altered landscape. Our results agree with other study systems where mate‐finding Allee effects occur, ranging from plants (Gascoigne et al., 2009) up to different animal taxa (Levitan, 2002; Stenglein & Van Deelen, 2016). Interestingly, simulated *T. graeca* populations become extinct under typical conditions in natural landscapes if sperm storage was limited to the same breeding season. However, simulated populations were viable with sperm storage of one year (except for the worst scenario of low initial densities in a human‐altered landscape). We also found that long sperm storage durability (i.e., 3–4 years) would be required to reduce the strength of the Allee effect and therefore the extinction risk under those conditions. Therefore, our study corroborates the hypotheses that sperm storage is an important trait of the reproductive strategy of our study species.

Given that, sperm storage is present in different taxa (Orr & Brennan, 2015), we suspect that our results should also hold in principle for other species that suffer mate limitation (Gascoigne et al., 2009). Female sperm storage has a number of advantages for some species’ reproductive strategy (Orr & Zuk, 2012). It lowers mating costs, ensures the fertilization of eggs despite asynchronous male and female reproductive cycles, and can increase the choice of sire (Orr & Brennan, 2015; Roques et al., 2006). However, negative effects, such as inbreeding and loss of genetic diversity, are likely to emerge in real populations when the connectivity between subpopulations is lost (Andersen, Fog, & Damgaard, 2004). Therefore, it is crucial to understand the effect of sperm storage on the mating dynamics, demography, and on aspects of the ecology of different species, especially those that are endangered or suffer human perturbations as landscape fragmentation (Anthonysamy et al., 2014; Keinath et al., 2017).

The sperm storage durability is extremely variable across taxa (from hours to years) (Holt & Fazeli, 2016). Some studies have estimated sperm viability lasting up to 3 years in tortoises (Cutuli et al., 2013), but this durability is unknown for many species with low movement ability (Pearse & Avise, 2001). Moreover, the effects of this variability on aspects of the ecology and mating systems have not yet been addressed among individuals of the same species (Orr & Brennan, 2015). Our results suggest that longer sperm storage durability may be especially useful for the populations that occur in patchy habitats with low population densities and show low movement ability (Walter et al., 2016). Given the potentially major importance of longer sperm storage for the biological fitness of species with limited movement ability, future studies may evaluate this trait from experiments run in captivity, in which females are isolated from males (e.g., Palmer, Rostal, Grumbles, & Mulvey, 1998). This knowledge could also be useful for improving captive breeding in the recovery of endangered species (Snyder et al., 1996).

Mate‐finding Allee effects may also frequently occur for populations in expansion, where densities that border the species range are typically low (Shaw, Kokko, & Neubert, 2018; Walter et al., 2016). We expect that a long sperm storage durability would be important for species conservation and to contribute to successful range shifts in a climate change scenario (Estrada, Morales‐Castilla, Caplat, & Early, 2016; Morrison, Estrada, & Early, 2018). Therefore, management actions may include measures to promote mate finding by, for example, increasing landscape connectivity (Beier, Majka, & Spencer, 2008). However, long sperm storage durability could also favor population growth and the spread of invasive species (Kanarek & Webb, 2010).

To conclude, our model STEPLAND synthesizes and extrapolates data and different pieces of information on movement and demography of the spur‐thighed tortoise *T. graeca*. Long‐term simulations of spatial population dynamics allowed us to evaluate the ecological implications of sperm storage durability, which is an essential trait of many species’ reproductive strategy. Overall, we found that prolonged sperm storage reduces the strength of the mate‐finding Allee effect in natural and human‐altered landscapes and especially under low initial population density scenarios. Conducting such long‐term experiments while manipulating this biological trait would be impossible in the field. Therefore, our results highlight the importance of modeling approaches for placing mechanisms, such as sperm storage, in a population dynamics context because they would require long study periods to become evident in the field (Stillman, Railsback, Giske, Berger, & Grimm, 2014).

## AUTHOR CONTRIBUTIONS

AG, PB, TW, EG, RCRC, and MVJF conceived the ideas. JDA, EG, TW, and AG developed the IBM. MVJF, ASA, RCRC, and EG analyzed the data. MVJF led the writing of the manuscript. All the authors contributed critically to the draft and gave their approval for its publication.

## Data Availability

Model name: STEPLAND. Availability: The full code of STEPLAND, its parameterization and the main results files are available in the figshare repository at https://doi.org/10.6084/m9.figshare.11498703.v1. Licence: Apache 2.0
