# Supplementary material for: Sperm storage reduces the strength of the mate‐finding Allee effect
Source: Ecol Evol. 2020 Feb 7;10(4):1938–48. doi: 10.1002/ece3.6019 (PMC7042743; doi:10.1002/ece3.6019)
Supplement: Supplementary file 1 [file ECE3-10-1938-s001.docx]

**APPENDIX S1**

**Sperm storage reduces the strength of the mate-finding Allee effect**

**Appendix S1.** Model parameterisation based on empirical data and bibliographic review on *Testudo graeca* and *T. hermanni* (a species with similar habitat requirements and biological characteristics to *T. graeca*). Parameters values were calculated as the average among the available. When more than three values were available, we discarded the extreme values and calculated the average. See Appendix S3 for a detailed description of the mortality, reproduction and movement processes.

Table S1. Parameters related to the process mortality.

| Parameter | Description | Values | Observations | References |
| --- | --- | --- | --- | --- |
| subadult_age | Age transition from immature to subadult classes | 4 years old |  | Sanz-Aguilar et al. (2011);  Ben Kaddour et al. (2005) |
| male_adult_age | Age transition of males from subadult to adult classes | 7 years old |  |  |
| female_adut_age | Age transition of females from subadult to adult classes | 7 years old |  |  |
| *S*_0_ | Survival rates of individuals with less than a year. Representing hatching rate and newborn survival. Calculated as their product. | 0.28 | Hatching rates: 0.824^1^ , 0.613^2^  Newborn survival rate: 0.39^3^ | ^1^Díaz-Paniagua, Keller & Andreu (1997);  ^2^Díaz-Paniagua, Andreu & Keller (2006);  ^3^Keller, Díaz-Paniagua & Andreu (1998) |
| *S*_immatures_ | Survival rates of immature tortoises (< subadult_age years). | 0.407 | *T. graeca:* 0.20^1^, 0.72^4^, 0.055^5^,  *T. hermanni:* 0.69^6^, 0.52^7^. | ^1^Sanz-Aguilar et al. (2011);  ^2^Rodriguez-Caro et al. (2013);  ^3^Rodríguez-Caro et al. (2019);  ^4^Díaz-Paniagua, Keller, and Andreu (2001)  ^5^Rouag et al. (2007)  ^6^Fernandez-Chacón et al. (2011)  ^7^Henry et al. (1998) |
| *S*_subadults_ | Survival rates of subadult tortoises (≥ subadult_age and < male_adult age or female_adult_age). | 0.863 | *T. graeca:* 0.79^1^, 0.845^2^  *T. hermanni*: 0.12^6^, 0.88^7^ |  |
| *S*_adults_ | Survival rates of adult tortoises (≥ male_adult_age or female_adult_age). | 0.98, 0.95 | *T. graeca:* 0.98^1^, 0.9843^2^, 0.9465^2^, 0.92^3^, 0.909^4^, 0.9606^5^  *T. hermanni:* 0.98^6^, 0.95^7^. |  |

**Note that the survival probability for each age class is 1- mortality rate.*

Table S2. Parameters related to the process reproduction.

| Parameter | Description | Values | Observations | References |
| --- | --- | --- | --- | --- |
| female_repro_age | Age at maturity of females | 10 years old |  | Rodríguez-Caro et al. (2013) |
| male_repro_age | Age at maturity of males | 7 years old |  | Rodríguez-Caro et al. (2013) |
| repro_female | Probability to lay eggs each clutching date | 0.66 |  | Rodríguez-Caro et al. (2014) |
| N_clutch | Number of clutches in spring. | 0, 1, 2 , 3 | It is 1 or higher when females have available sperm. The mean value considering the probability to lay eggs is 1.98. | Díaz-Paniagua et al. (1996), (1997); unpublished data |
| DIST | Threshold of maximal distance among females and males that allows mating. | 500 m | Mate-finding efficiency | Evaluated and fixed by a previous sensitivity analysis (Graciá et al., 2020). |
| sperm_cad | Time of caducity of the stored sperm. | 0, 1, 2, 3, 4 years | Females store sperm at least during one breeding season up to 3 or 4 years | Roques et al. (2006);  Cutuli et al. (2013);  Parameter tested in this study. (Jiménez-Franco et al., 2020). |
| clutch_size | Probability of the number of eggs per clutch in each clutching date. | 1 egg = 0.0677  2 eggs = 0.1594  3 eggs = 0.4262  4 eggs = 0.2590  5 eggs = 0.0717  6 eggs = 0.0040  7 eggs = 0.0120 | Calculated from more than 500 radiographs in field. See Figure S4 in Appendix S3. | Rodríguez-Caro et al. (2014) |
| sex ratio | The ratio of male to females in a population | 0.5 | The majority of *T. graeca* populations show balanced sex ratios | Graciá et al. (2017) |
|  |  |  |  |  |

Table S3. Parameters related to the process movement.

| Parameter | Description | Values | Observations | References |
| --- | --- | --- | --- | --- |
| AGE | Age transition to achieve home ranges over 100 m^2^ | 4 years |  | Keller, Díaz-Paniagua & Andreu (1997).  Evaluated and fixed by a sensitivity analysis (Graciá et al., 2020) |
| SITE | Stability of home ranges. Site fidelity among years is emulated maintaining the location of the focal-point attractor of tortoises for their whole life (“YES”). | YES |  | Evaluated and fixed by a sensitivity analysis (Graciá et al., 2020) |
| PERSON | Behavioural plasticity. Stable individual personalities are emulated maintaining individual movement parameterisation through the whole life of tortoises (“YES”). | YES | Stable individual personalities are emulated maintaining individual movement parameterisation through the whole life of tortoises (“YES”). | Golubović et al. 2014, 2017  Evaluated and fixed by a sensitivity analysis (Graciá et al., 2020) |
| PMOV | Probability of moving during a given day, being dependent of the month and the sex of the tortoise | See Supporting Information Appendix S3 | See Figure S4 in Appendix S3 | Anadón et al. (2012) |
| DMOV | Discrete probability to move *s* cell steps during each movement step | See Supporting Information Appendix S3 | See Figure S4 in Appendix S3 | Anadón et al. (2012) |
| AU1 | Autocorrelation between consecutive movement steps, affecting only the first cell step of each movement step | See Supporting Information Appendix S3 | See Figure S5 in Appendix S3 | Anadón et al. (2012) |
| AU2 | Autocorrelation between cell steps | See Supporting Information Appendix S3 | See Figure S5 in Appendix S3 | Anadón et al. (2012) |
| dHB | Distance threshold below which no homing behaviour occurs | See Supporting Information Appendix S3 | See Figure S6 in Appendix S3 | Anadón et al. (2012) |
| rHB | Temporal delay (in days) of the homing behaviour since *dHB* was exceeded | See Supporting Information Appendix S3 | See Figure S6 in Appendix S3 | Anadón et al. (2012) |
| H1W | Habitat-dependent weights of each cell to be occupied of intensive land uses | See Supporting Information Appendix S3 | See Figure S7 in Appendix S3 | Anadón et al. (2012) |
| H2W | Habitat-dependent weights of each cell to be occupied of traditional agriculture | See Supporting Information Appendix S3 | See Figure S7 in Appendix S3 | Anadón et al. (2012) |
| H3W | Habitat-dependent weights of each cell to be occupied of flat natural areas | See Supporting Information Appendix S3 | See Figure S7 in Appendix S3 | Anadón et al. (2012) |
| H4W | Habitat-dependent weights of each cell to be occupied of natural areas on slope | See Supporting Information Appendix S3 | See Figure S7 in Appendix S3 | Anadón et al. (2012) |
| H5W | Habitat-dependent weights of each cell to be occupied of non-permeable infrastructures | See Supporting Information Appendix S3 | See Figure S7 in Appendix S3 | Anadón et al. (2012) |
|  |  |  |  |  |

**LITERATURE CITED**

Anadón, J. D., Wiegand, T., & Giménez, A. (2012). Individual‐based movement models reveals sex‐biased effects of landscape fragmentation on animal movement. *Ecosphere*, 3, 1-32.

Ben Kaddour, K., El Mouden, E. H., Slimani, T., Lagarde, F., & Bonnet, X. (2005). Sexual dimorphism, growth and maturation patterns of Testudo g. graeca, in the Central Jbilets, Morocco. Revue D Ecologie-La Terre Et La Vie, 60, 265-278.

Cutuli, G., Cannicci, S., Vannini, M., & Fratini, S. (2013). Influence of mating order on courtship displays and stored sperm utilization in Hermann's tortoises (Testudo hermanni hermanni). *Behavioral Ecology and Sociobiology*, 67, 273-281.

Díaz-Paniagua, C., Andreu, A. C., & Keller, C. (2006). Effects of temperature on hatching success in field incubating nests of spur-thighed tortoises, Testudo graeca. *The Herpetological Journal*, 16, 249-257.

Díaz‐Paniagua, C., Keller, C., & Andreu, A. C. (1997). Hatching success, delay of emergence and hatchling biometry of the spur‐thighed tortoise, Testudo graeca, in south‐western Spain. *Journal of Zoology*, 243, 543-553.

Díaz‐Paniagua, C., Keller, C., & Andreu, A. C. (2001). Long‐term demographic fluctuations of the spur‐thighed tortoise Testudo graeca in SW Spain. *Ecography*, 24, 707-721.

Fernández‐Chacón, A., Bertolero, A., Amengual, A., Tavecchia, G., Homar, V., & Oro, D. (2011). Spatial heterogeneity in the effects of climate change on the population dynamics of a Mediterranean tortoise. *Global Change Biology*, 17, 3075-3088.

Golubović, A., Anđelković, M., Arsovski, D., Bonnet, X., & Tomović, L. (2017). Locomotor performances reflect habitat constraints in an armoured species. Behavioral Ecology and Sociobiology, 71(6), 93.

Golubović, A., Andjelković, M., Arsovski, D., Vujović, A., Iković, V. Djordjević, S., & Tomović, L. (2014). Skills or strength—how tortoises cope with dense vegetation?. Acta Ethologica, 17(3), 141-147.

Graciá, E., Rodríguez-Caro, R.C., Andreu, A.C., Fritz, U., Giménez, A., Botella, F. (2017) Human-mediated secondary contact of two tortoise lineages results in sex-biased introgression. Scientific Reports, 7, 4019. http://www.nature.com/articles/s41598-017-04208-4

Graciá, E., Rodríguez-Caro, R.C., Sanz-Aguilar, A., Anadón, J.D., Botella, F., García-García, A.L., Wiegand, T., Giménez, A. (2020). Assessment of the key evolutionary traits that prevent extinctions in human-altered habitats using a spatially explicit individual-based model. *Ecological modelling*. 415, 108823.

Henry, P. Y., Nougarède, J. P., Pradel, R., & Cheylan, M. (1998). Survival rates and demography of the Hermann’s tortoise *Testudo* hermanni in Corsica, France. In: Miaud, C., Guyétant, R. (Eds.), Current Studies in Herpetology. Societas Europaea Herpetologica, Le Bourget du Lac, France, pp. 189–196.

Jiménez-Franco, M. V., Giménez, A., Rodríguez-Caro, R., Sanz-Aguilar, A., Botella, F., Anadón, J. D., Wiegand, T., & Graciá, E. (2020). Data from: Sperm storage reduces the strength of the mate-finding Allee effect. FigShare Repository. Software. https://doi.org/10.6084/m9.figshare.11498703.v1

Keller, C., Díaz-Paniagua, C., & Andreu, A. C. (1997). Post-emergent field activity and growth rates of hatchling spur-thighed tortoises, Testudo graeca. *Canadian Journal of Zoology*, 75, 1089-1098.

Keller, C., Díaz-Paniagua, C., & Andreu, A. C. (1998). Survival rates and causes of mortality of Testudo graeca hatchlings in southwestern Spain. *Journal of Herpetology*, 238-243.

Rodríguez-Caro, R. C., Graciá, E., Anadón, J. D., & Gimenez, A. (2013). Maintained effects of fire on individual growth and survival rates in a spur-thighed tortoise population. *European Journal of Wildlife Research*, 59, 911-913.

Rodríguez-Caro, R. C., White, E. R., Wiegand, T., Sanz-Aguilar, A., Giménez, A., Graciá, E., van Benthem, K.J., & Anadón, J. D. (2019). A low cost approach to estimate demographic rates using inverse modelling. 237: 358-365

Rodríguez-Caro, R. C., Graciá, E., Anadón, J. D., Botella, P., & Giménez, A. (2014). A compensatory response in reproduction of the tortoise *Testudo graeca* against habitat disturbances?. XIII Congreso Luso-Español de Herpetología. Aveiro (Portugal).

Roques, S., C. Díaz-Paniagua, & A. C. Andreu. (2004). Microsatellite markers reveal multiple paternity and sperm storage in the Mediterranean spurthighed tortoise, Testudo graeca. *Canadian Journal of Zoology*, 82: 153-159.

Rouag, R., Benyacoub, S., Luiselli, L., Mouden, E. H. E., Tiar, G., & Ferrah, C. (2007). Population structure and demography of an Algerian population of the Moorish tortoise, *Testudo graeca*. *Animal Biology*, *57*, 267–279.

Sanz-Aguilar, A., Anadón, J.D., Giménez, A., Ballestar, R., Graciá, E., & Oro, D. (2011). Coexisting with fire: The case of the terrestrial tortoise *Testudo graeca* in mediterranean shrublands. *Biological Conservation*, 144, 1040–1049.
