# Supplementary material for: Sperm storage reduces the strength of the mate‐finding Allee effect
Source: Ecol Evol. 2020 Feb 7;10(4):1938–48. doi: 10.1002/ece3.6019 (PMC7042743; doi:10.1002/ece3.6019)
Supplement: Supplementary file 2 [file ECE3-10-1938-s002.docx]

**APPENDIX S2**

**Sperm storage reduces the strength of the mate-finding Allee effect**

**Script in R for the calculation of stable age distribution.**

*rm(list=ls(all=TRUE))  

############################################################################## FEMALE based Matrix Model for Testudo graeca
#############################################################################*

*library(MASS)
library(popbio)

# Mean of demographic parameters (see details in Appendix S1)
s1 <- 0.497        #  Immature survival (1-3yrs). 1-* Mortality rates of immature tortoises *s2 <- 0.863        #  Subadult survival (4-6 years). 1-* Mortality rates of subadult tortoises *s3 <- 0.954     #  Adult survival (≥7years). 1-* Mortality rates of adult tortoises *NC<- 2            ### Number of clutches
CS<- 3.0482      ### Number of eggs.
ES<- 0.2802        ### Hatching success*1st winter survival
SR<- 0.5         ### Sex ratio
MP<- 1       ### Mating probability*

*RR<- NC*CS*ES*MP ### Reproductive rate*

*# Pre birth-pulse matrix model for Testudo graeca

A <- matrix(
c(0,    0,    0,    0,    0,    0,    0,    0,    0,    RR*SR,
s1,    0,    0,    0,    0,    0,    0,    0,    0,    0,
0,    s1,    0,    0,    0,    0,    0,    0,    0,    0,
0,    0,    s1,    0,    0,    0,    0,    0,    0,    0,
0,    0,    0,    s2,    0,    0,    0,    0,    0,    0,
0,    0,    0,    0,    s2,    0,    0,    0,    0,    0,
0,    0,    0,    0,    0,    s2,    0,    0,    0,    0,
0,    0,    0,    0,    0,    0,    s3,    0,    0,    0,
0,    0,    0,    0,    0,    0,    0,    s3,    0,    0,
0,    0,    0,    0,    0,    0,    0,    0,    s3,    s3),
nrow = 10, byrow = TRUE)
 
# Popultion growth rate (lambda= 1.008), the dominant eigenvalue of A*

lambda(A)

# *Stage of stable distribution (note that last value is for individuals with age ≥10 years),
# the right eigenvector of A*

stable.stage(A)

Note that the deterministic expected reproductive rate *RR_det_* of the population is given by *NC*CS*ES*MP* = 1.71.
