# Supplementary material for: Sperm storage reduces the strength of the mate‐finding Allee effect
Source: Ecol Evol. 2020 Feb 7;10(4):1938–48. doi: 10.1002/ece3.6019 (PMC7042743; doi:10.1002/ece3.6019)
Supplement: Supplementary file 3 [file ECE3-10-1938-s003.doc]

**APPENDIX S3**

**Sperm storage reduces the strength of the mate-finding Allee effect**

**Overview, Design concepts, and Details protocol of the individual-based model used in this study (ODD).**

**STEPLAND: a spatially explicit individual-based model to simulate spur-thighed populations’ viability**

**Purpose**

The main objective of STEPLAND is to investigate the effects of biological traits, population characteristics (e.g., adult survival) and the habitat landscape, on population viability over a timespan of some hundreds of years (Graciá et al., 2020). The model was implemented into Python 2.7 and its code, parameterisation and the main results files are available in the FigShare repository (Jiménez-Franco et al., 2020; <https://doi.org/10.6084/m9.figshare.11498703.v1>).

**Entities, attributes and spatial scales**

STEPLAND comprises two different entities: landscapes and individuals. landscapes comprise an area of 3 km × 3 km composed of a 10 m × 10 m grid of cells (although other areas and grids are also possible). Each cell is characterised by its position (x and y coordinates) and its habitat category:

H1: intensive land use

H2: traditional agriculture land

H3: natural flat areas

H4: natural areas on slopes

H5: non-permeable infrastructures

These categories were taken from a previous study that investigated the effects of habitat loss and fragmentation on tortoise movements (Anadón, Wiegand, & Giménez, 2012). Landscapes are enclosed by a non-permeable border and host closed and isolated populations (Fig. S1).

Tortoises are characterised by the following attributes: gender, age, time for stored sperm caducity, their actual location and past locations. Additionally, the nine parameters AU1, AU2, dHB, rHB, H1W, H2W, H3W, H4W and H5W govern their movement and the location of their focal-point attractor. These nine parameters can be maintained for their whole lifetimes. Except for H5W, these parameters were taken from the movement analysis of Anadón et al. (2012). See the description of the movement process for further information.


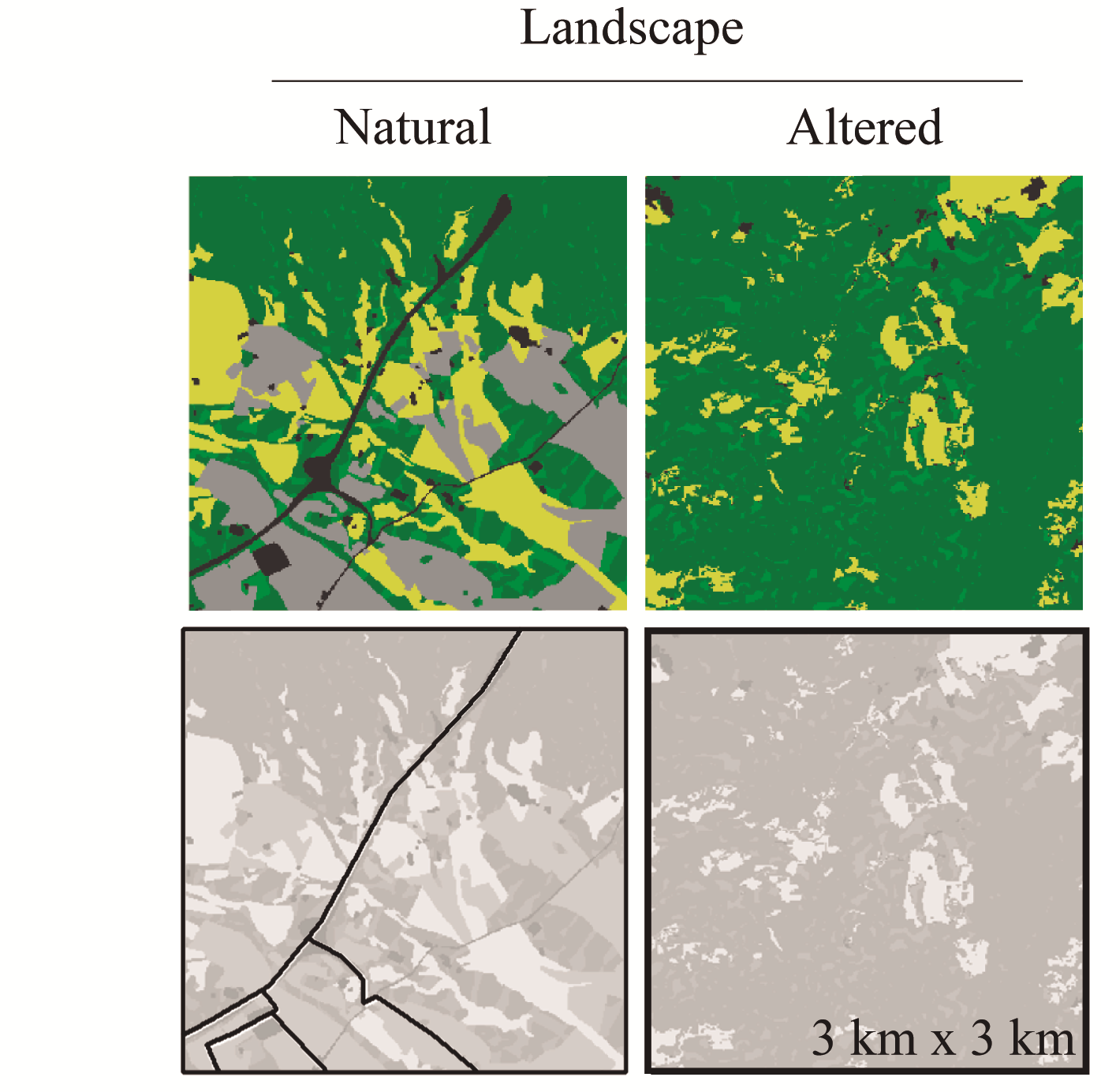

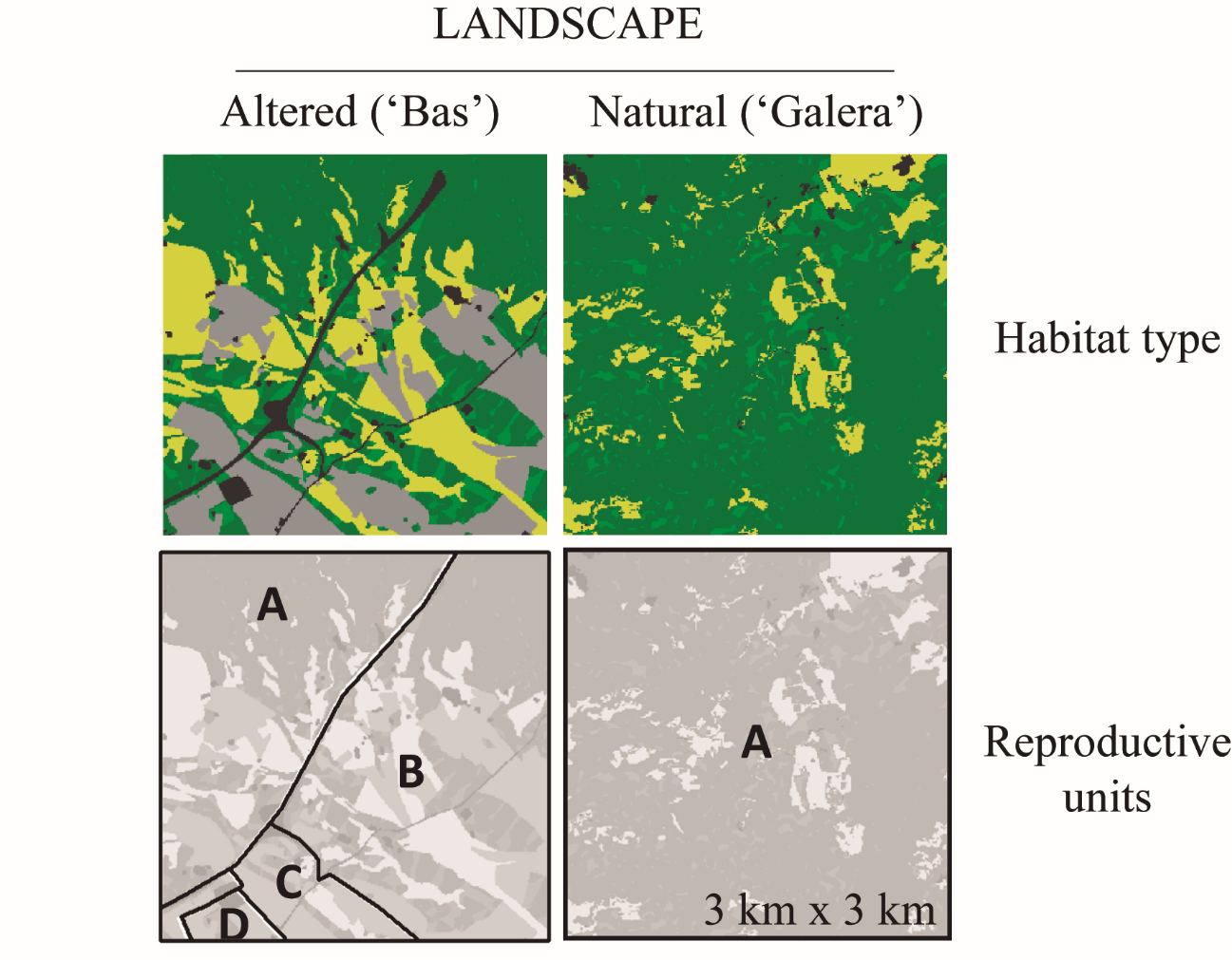


**Figure S1.** Examples of the landscapes that may be used in STEPLAND. Habitat categories include: non-permeable infrastructures (black), intensive land use (grey), traditional agriculture lands (yellow), natural flat areas (light green) and natural areas on slopes (dark green). Grey landscapes show the barrier effects of landscape features for the mating submodel. The natural landscape lacks internal barriers, but roads and intensive agriculture divide the human-altered landscape.

**Input, initialisation and output**

Inputs include the landscape, the initial population and model parameterisation; they are all included in an SQL database that is directly read by the model. Initially, *N*_0_ =180 or 90 tortoises are randomly distributed within the landscape, which represents the medium and low densities that are typically found in the natural species populations in SE Spain (Anadón et al., 2009). Note that the initial populations (individuals and their locations) were the same in all the simulation scenarios that started with the same *N*_0_. To avoid biases caused by extreme spacing of the initial locations of individuals, we tested 15 different sets of locations and selected for our study a set with mean minimum Euclidean distances between females and males close to the mean obtained from the other simulations (Table S1). Each parameterisation was then simulated 64 times in a given landscape using the same initial population.

**Table S1.** Test for biases in extinction probabilities after 200 years due to individuals’ initial locations. We tested 15 sets of locations (5 per initial density) in the natural landscape with a standard parameterisation (Appendix S1). For our study, three sets of locations with mean minimum Euclidean distances between males and females close to the obtained means from the tested simulations were selected.

| Initial density | Mean extinction probability ± SD | Mean min. fem-male distance ± SD (m) in the tested sets of the initial locations | Mean min. fem-male distance ± SD in the selected set of initial locations |
| --- | --- | --- | --- |
| *N*_0_=90 | 0.06±0.07 | 422.5±142.2 | 417.4±150.62 |
| *N*_0_=180 | 0±0 | 295.9±38.8 | 338.6±169.19 |

The different age classes of the initial population follow a stable age distribution predicted by an age-stage structured deterministic matrix population model (Caswell, 2001) that was derived using the POPBIO package in the R software (Stubben & Milligan, 2007; R Core Team, 2014; R scripts are shown in Appendix S2). The demographic parameters of the matrix model are the same as the demographic parameters of the simulation model, but the growth rate predicted by the deterministic matrix model does not consider demographic stochasticity and mate-finding Allee effects (Appendix S2). The complete model parameterisation is shown in Appendix S1.

Data output consists of CSV files that comprise the surviving tortoises of the population and their attributes (location in the landscape, gender and age) for every 10 years during the simulated years (Jiménez-Franco et al., 2020). Own scripts, developed in R (R Core Team, 2016) and Python 2.7, allow these massive outputs to be processed. For operational reasons, the simulation stops if the total number of tortoises in the landscape exceeds 4,500.

**Temporal scales, process overview and scheduling**

In STEPLAND, each movement step of individual represents 1 day, while outputs are summarised for 10-year periods. All the simulations were run for 200 years (corresponding to approximately eight tortoise generations).

Tortoises are subject to the basic processes movement, reproduction, mortality and aging. They can move up to 4 times a day according to their attributes and movement parameters, the time of the year and the habitat category of surrounding cells (as modelled and parameterised by Anadón et al. 2012). Adult females can reproduce up to 3 times/year, as evidenced by own data and previous studies that have addressed the reproduction of this species (Díaz-Paniagua, Keller, & Andreu, 1996; 1997). However, spatial effects may hinder mating, including a too large distances between females and their nearest adult males, or presence of non-permeable barriers between them. Annual mortality rates are implemented through a single event of mortality at the end of the year. The age of surviving tortoises is then updated, and their movement attributes may also be updated (i.e., in some scenarios the last location of the year may be used as their focal point attractor; see movement submodel for further information). Figure S2 provides a general overview of the model and the following paragraphs describe the main processes in more detail.


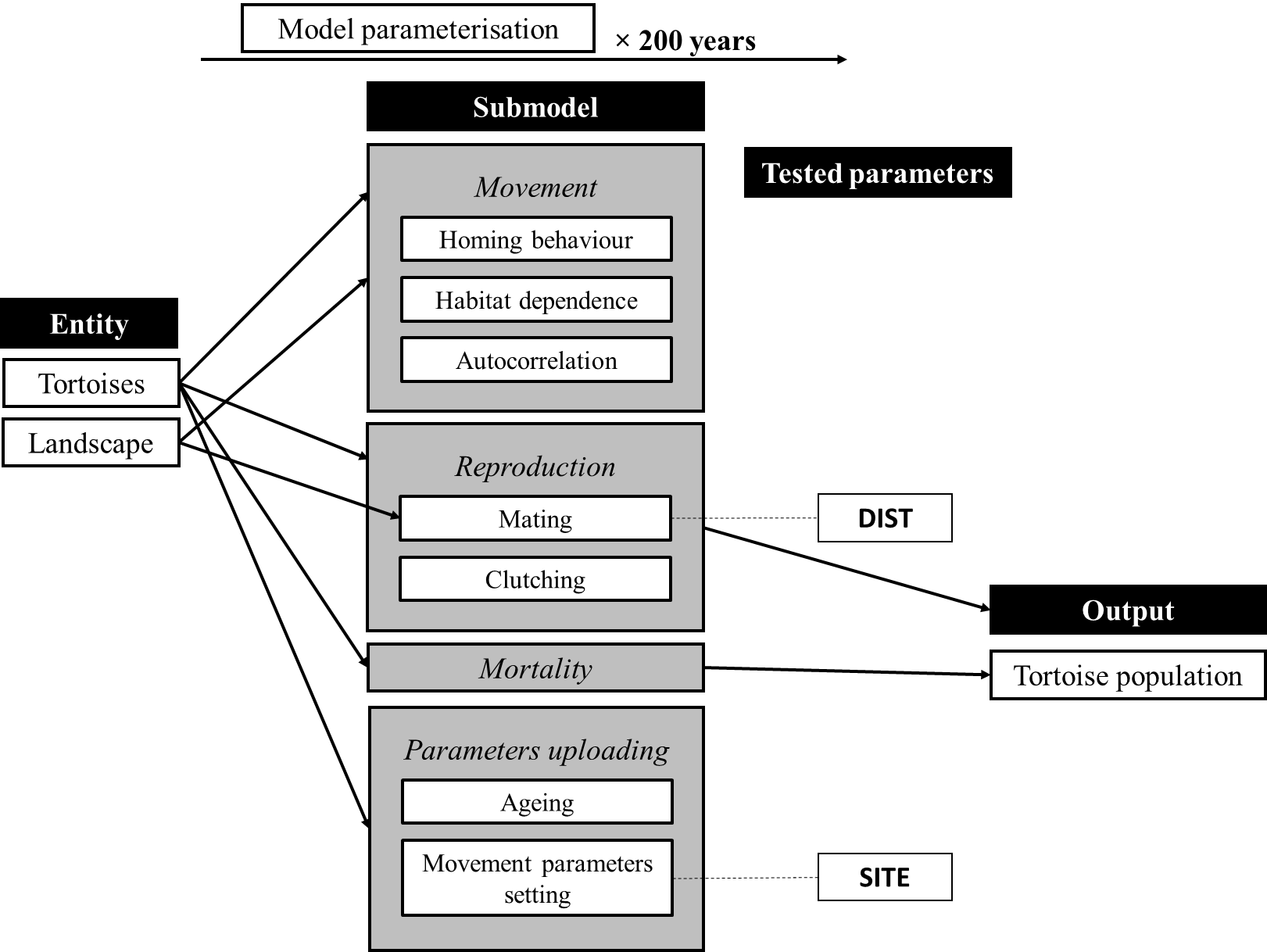


**Figure S2.** Main processes implemented in STEPLAND. Inputs are the landscape and the initial tortoise population. During the simulation, the individuals are subject to the basic processes: movement, reproduction, mortality and aging; and may be subject to parameter updates (e.g., variations in the location of their focal point attractor). In this study, we simulated population dynamics for 200 years and evaluated the sensitivity of extinction rates to variations in the SITE and DIST parameters.

*Movement*

STEPLAND includes the movement model developed and parameterised by Anadón et al. (2012). They used individual-based modelling and pattern-oriented modelling to parameterise the movement model based on radio-tracking data. One main result of Anadon et al. (2012) is that land-use intensification in SE Spain had a strong impact on spur-thighed tortoises’ movement and behaviour. While tortoises showed high site fidelity and slight individual variability in natural landscapes, movement and behaviour varied strongly among individuals in human-altered landscapes. Particularly for females, a wide range of movement patterns was found, which ranged from strong home behaviour to unbounded movements. Anadón et al. (2012) determined a total of 5,627 individual parameterisations of annual movements that were compatible with the radio-tracking data (Table S2). Females in human-altered landscapes formed the largest set of individual movement parameterisations (Table S3).

**Table S2.** Subset as an example of the parameterisations assigned to individuals for their movements as determined by Anadón et al. (2012). In our simulations, these parameterisations were randomly assigned to the initial individuals and to the newborns (depending on sex and landscape type).

| Sex and habitat type | AU1 | AU2 | dHB | rHB | Habitat-dependent weights for cells | | | | |
| --- | --- | --- | --- | --- | --- | --- | --- | --- | --- |
|  |  |  |  |  | H1W | H2W | H3W | H4W | H5W |
| male_natural | 0.05 | 0.7 | 50 | 25 | 0.1 | 5 | 5 | 8 | 0 |
| male_natural | 0.05 | 0.65 | 75 | 3 | 0.1 | 20 | 11 | 3 | 0 |
| male_natural | 0.75 | 0.75 | 25 | 10 | 0.1 | 15 | 2 | 13 | 0 |
| female_natural | 0.1 | 0.25 | 50 | 7 | 0.1 | 11 | 17 | 10 | 0 |
| female_natural | 0.05 | 0.3 | 50 | 10 | 0.1 | 5 | 2 | 7 | 0 |
| female_natural | 0.15 | 0.9 | 25 | 10 | 0.1 | 17 | 5 | 13 | 0 |
| male_alterned | 0.7 | 0.85 | 75 | 3 | 0.1 | 5 | 8 | 18 | 0 |
| male_alterned | 0.65 | 0.65 | 100 | 10 | 0.1 | 5 | 15 | 6 | 0 |
| male_alterned | 0.25 | 0.5 | 75 | 3 | 0.1 | 4 | 13 | 10 | 0 |
| female_alterned | 0.55 | 0.85 | 175 | 60 | 0.1 | 9 | 12 | 2 | 0 |
| female_alterned | 0.25 | 0.65 | 10000 | 30 | 0.1 | 8 | 16 | 1 | 0 |
| female_alterned | 0.25 | 0.85 | 500 | 4 | 0.1 | 15 | 10 | 3 | 0 |

AU1: autocorrelation between consecutive movement steps; AU2: autocorrelation between cell steps; dHB; distance threshold below which no homing behaviour occurs rHB: temporal delay (in days) of homing behaviour as dHB was exceeded; H1W: weight for cells with intensive land use: H2W: weight for cells with traditional agriculture; weight for cells with H3W: natural flat areas; weight for cells with H4W: natural areas on slopes; weight for cells with H5W: non-permeable infrastructures.

**Table S3.** Number of individual parameterisations for the movement process, as determined by Anadón et al. (2012).

|  | Natural landscape | Altered landscape |
| --- | --- | --- |
| Males | 139 | 51 |
| Females | 155 | 5,282 |

Tortoises older than the age threshold given by the parameter age_first_mov, can change their location up to 4 times/day (i.e., four movement steps; Fig. S3). The active period of one day is divided into four 2- to 3-hour periods, and each of these periods a random trial is made with the PMOV parameter being the probability of moving during a period. PMOV is dependent of the month and tortoise gender, for example, tortoises are inactive during their hibernation and aestivation periods (Fig. S4a). If the PMOV trial indicated movement, DMOV is the discrete probability of moving *s* cell steps in each movement step (“cell-to-cell movements”; Fig. S3). DMOV varies between adult males and females (Fig. S4b). It was also assumed that subadults have a reduced movement capability than adults. To implement this, the number of cell-to-cell steps in DMOV was assumed to increase gradually with age until the reproductive age:

DMOV_subadult_=round(DMOV_adult_×(subadult's age)/(reproductive age)) (1)


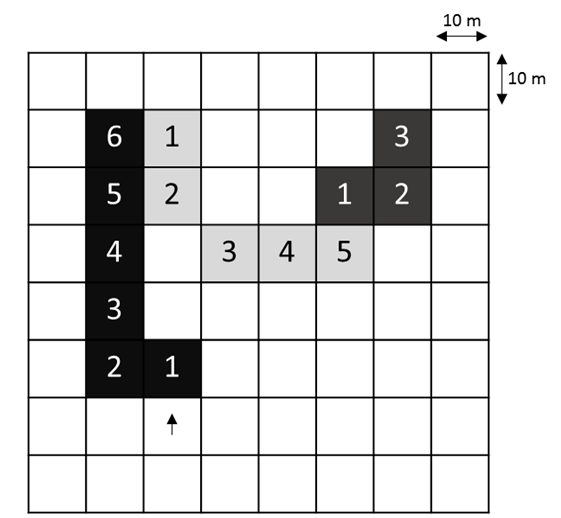


**Figure S3.** Example of the simulated movement of a tortoise during a given day. Starting from the arrow, the tortoise takes three movement steps during the day (represented by different colours), with six, five and three cell-to-cell steps each (movement directions are represented by numbers).


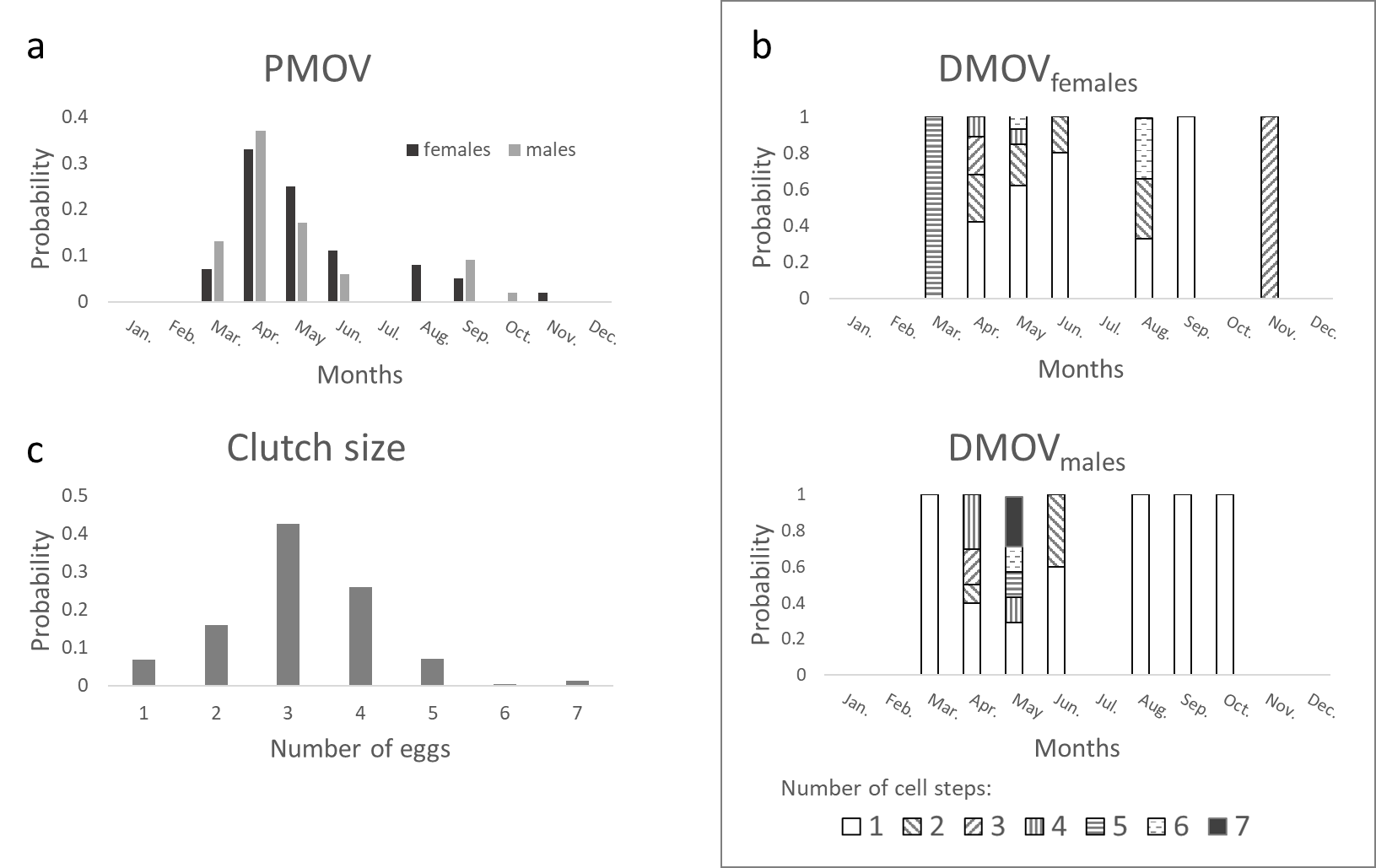


**Figure S4.** Discrete probability distributions of the parameters PMOV (a); DMOV of females and males (b) and clutch_size (c).

In each cell-to-cell movement step, three submodels interact in the selection of one of the eight neighbouring cells surrounding the tortoise by determining their associated weights, which are then multiplied to calculate its final probability to be occupied:

1. *Autocorrelation*: describes the tendency of keeping the previous direction. The eight cells surrounding the present location are numbered from *i* = 1, …, 8, where the index *i* = 1 indicates the cell-by-cell step following exactly the previous direction. Then *i* = 2, …, 8 are numbered clockwise. The weights *d_i_* of the neighbouring cells with indices *i* = 1, …, 5 (movement to the right) are calculated as:

*d_i_*=max[0, (1.5+(0.5-i )×AU)/(1.5-0.5×AU)] (2)

For the remaining weights that represent movement to the left, isotropic movement (i.e., *d*_6_ = *d*_4_, *d*_7_ = *d*_3_, *d*_8_ = *d*_2_) is assumed. Finally, weights *d_i_* are normalised to add up to 1. As cell-to-cell steps are nested inside movement steps, two parameters were used to describe autocorrelation in movement. The AU1 parameter represents the autocorrelation between consecutive movement steps and affects only the first cell-to-cell step of each movement step. The AU2 parameter is the autocorrelation between the following cell-to-cell steps. Thus for the first cell-to-cell step, AU1, and AU2 were used for the other cell steps (Fig. S5).


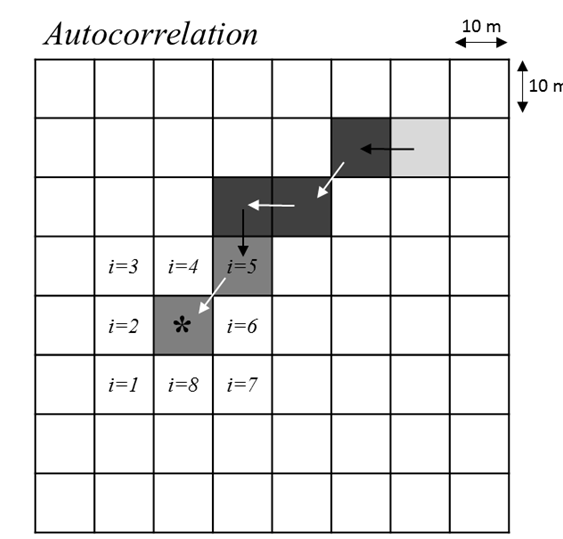


**Figure S5.** Schematic representation of the autocorrelation submodel. Arrows differentiate steps or cell step transitions using parameters AU1 (in black) or AU2 (in white).

1. *Homing behaviour:* modelled as the tendency of an individual to maintain a stable home range around a focal point attractor. Weights *b_i_* are numbered in the same way as the weights for autocorrelation. We used the first location of the individual as the focal-point attractor. To model homing behaviour, two critical distances were assumed around the focal point. *D* is the individual’s distance to its focal-point attractor and the *dHB* parameter is then the distance threshold below which no homing behaviour occurs. Firstly, if the individual is located close to the focal point, equal weights for the different directions are given (i.e., *b_i_* = 1/8 for all *i* = 1,.. 8, when *D* < *dHB*). Thus, if the individual is close to its focal point attractor, no homing behaviours occurs. Secondly, if the individual is located farther away than distance 2**dHB* from the focal point attractor, the bias towards the focal point is maximal and the cell *j* closest to the focal-point attractor has a probability of *b_j_* = 1 of being occupied, while the remaining seven cells have probability of *b_i_* = 0. In intermediate situations in which *dHB* < *D* < 2*dHB*, the bias of *b_j_* increases from 1/8 proportionally with distance *D* up to *2*d*HB with the formula:

*b_j_* = (*D-dHB*)/(*dHB*) and *b_i_* = (1 – *b_j_*)/7 for *i* ≠ *j* (3)

In order to enhance the model’s biological realism, a temporal delay was considered so that homing behaviour came into force after exceeding the distance threshold *dHB* from the focal point. This allows tortoises to temporally leave their most regularly used areas to meet specific needs, such as egg-laying in females or mate-searching in males (Díaz-Paniagua, Keller, & Andreu, 1995; Pérez, Giménez, Anadón, Martínez, & Esteve, 2002). The *rHB* parameter is the temporal delay (in days) of homing behaviour as *dHB* was exceeded (*R*, in days) (Fig. S6).


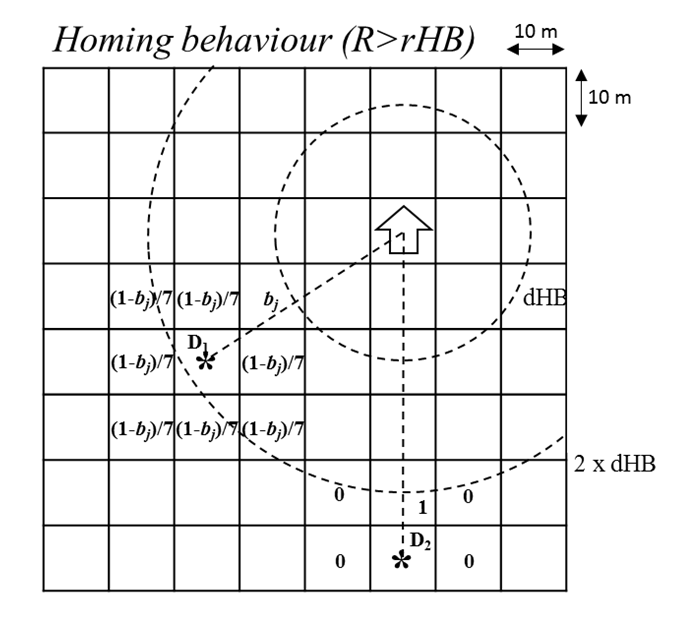


**Figure S6.** Schematic representation of the homing behaviour submodel. Asterisks indicate the location of tortoises, with distances *D*_1_ and *D*_2_ to the focal point attractor. NB: *dHB* < *D_1_* < 2*dHB* and *D_2_* > 2*dHB.*

1. *Habitat dependence:* in relation to habitat quality, the eight neighbouring cells receive habitat-dependent weights to be occupied through parameters H1W (intensive land use), H2W (traditional agriculture), H3W (natural flat areas), H4W (natural areas on slopes) and H5W (non-permeable infrastructures). The weights of the eight cells are subsequently normalised to add up to 1 (Fig. S7).


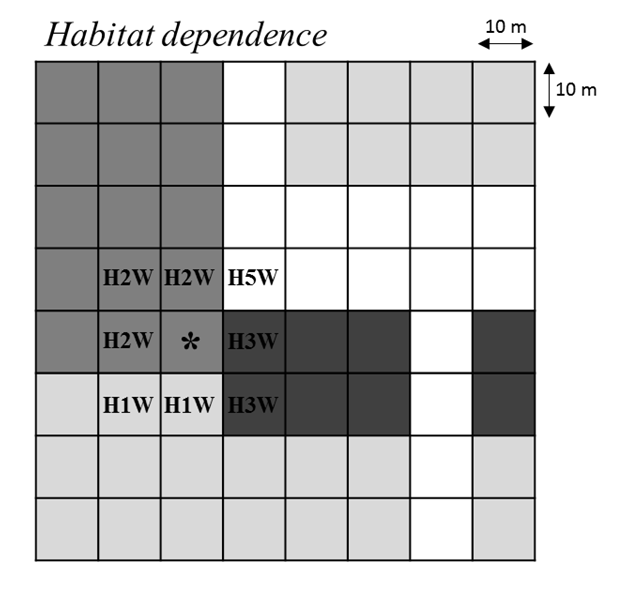


**Figure S7.-** Schematic representation of the habitat dependence submodel. Cell colours represent habitat categories.

*Reproduction*

Only adult individuals (aged ≥ 7 years for males and ≥ 10 years for females) reproduce (Sanz-Aguilar et al., 2011; Rodríguez-Caro, Graciá, Anadón, & Giménez, 2013). Sperm storage, a vital trait of spur-thighed tortoises (Roques, Díaz-Paniagua, & Andreu, 2004), was modelled as a temporal dissociation between mating and clutching.

1. *Mating*: once a year at the beginning of spring, mature females localise mature males in their surroundings (both with ages over female_repro_age and male_repro_age in Appendix S1). Given the spatial constraints to find mates, mating was assumed to occur only if the Euclidean distance between the female and the closest male was (at the time scheduled for mating) below a minimal distance given by the DIST parameter. In this study we selected DIST = 500m. This value was based on (i) a sensitivity analysis of Graciá et al. (2020) that evaluated the effect of DIST values between 100 m and 700 m on population dynamics; and (ii) mean maximal annual displacement distances of radiotracked *T. graeca* males and females (189-275 m and 148-271 m, respectively; Anadón et al. 2012). Moreover, there should be no barrier effects of the landscape between males and females (e.g. main roads or dense human infrastructures) in order to mate (Fig. S1). Some studies have evidenced that sperm can remain viable in the oviducts of females for as long as 3-4 years (Cutuli, Cannicci, Vannini, & Fratini, 2013), but no long-term studies have addressed a drop in sperm viability over time in-depth. Therefore, sperm caducity was modelled simply: females that find males can reproduce for 0, 1, 2, 3 and 4 years (represented by the sperm_cad parameter) without further mating, where 0 year represents the sperm stored during the same breeding season, and *n* year means reproduction for *n*+1 consecutive spring breeding seasons. If females find mates, then the stored sperm caducity timer is reset to 0, otherwise, a value of 1 is added to the sperm-caducity timer. Females can reproduce only if their sperm-caducity timer is below the value assigned for each simulation durability*.* For the scenarios with *n* = 0, sperm is only viable during the current breeding season, whereas the scenario with *n* = 4 allows females to reproduce for the next 4 years after mating. To be conservative, we assumed that sperm viability was 100% within the next *n* years, but 0% afterwards.
2. *Clutching*: after mating, the repro_female parameter is the probability that females with available sperm will be gravid when clutching can take place between one to three clutches per year, as in real populations (Díaz-Paniagua et al. 1996, 1997; unpublished data). The eggs were placed at the location of the female in spring and early summer when clutching occurs (from the end of April to the end of July), and the new individuals emerge from this position. The number of eggs that gravid females lay is determined from the discrete probability distribution clutch_size (Fig. S4c; Table S2 in Appendix S1). Clutch viability and new-born survival is later simulated by applying survival rates (Díaz-Paniagua et al. 1997, Díaz-Paniagua, Andreu, & Keller, 2006; see the next paragraph and Appendix S1 for further details regarding model parameterisation).

*Mortality*

A single mortality event occurs at the end of each simulated year. Annual survival rates vary among age classes, including new-borns (*S*_0_; representing hatching success and survivals of individuals below 1 year), immature individuals (*S*_immatures_; aged 1-3), subadults (*S*_subadults_; aged 4-6), and adults (*S*_adults_; aged ≥7) (Díaz-Paniagua, Keller, & Andreu, 2001; 2002, Sanz-Aguilar et al., 2011). New-borns are represented as individuals whose age equals 0, while age cuttings apply (subadult_age, female_adut_age and male_adult_age) for the transition among the remaining classes (Table S1 in Appendix S1). The individuals (and their attributes) that died disappear from the population.

*Parameters uploading*

1. *Ageing:* the age of each living individual sums 1 at the end of the year.

**Design concepts**

*Basic principles*: the model assumes that: i) tortoises maintain age-class transitions and survival rates in human-altered landscapes; ii) habitat loss and fragmentation cause changes in their movement and behaviour (as reported by Anadón et al. 2012); iii) non-permeable barriers hinder tortoise mating.

*Emergence:* simulations may result in different population dynamics due to variations in tortoises’ reproductive success.

*Adaptation:* tortoises face fragmentation by modifying their movement decisions. They avoid intensive land uses and non-permeable infrastructures.

*Objective:* tortoises do not have any particular objective implemented into simulations but, as previously mentioned, they modify their movement decisions according to habitat characteristics.*Sensing:* tortoises are able to detect habitat loss and fragmentation in landscapes of 3 km × 3 km (i.e., natural vs. human-altered landscapes). They also recognise their surrounding habitat in 30 m × 30 m (the eight adjacent grid cells).

*Interaction:* tortoises are able to mate if they are separated by a distance shorter than that given by the DIST parameter and if there are no geographical barriers between them.

*Learning:* tortoises do not change their behaviour according to their experience.

*Prediction:* tortoises do not have the capacity to predict future conditions.

*Stochasticity:* tortoises’ initial location, and assigning movement parameters to them, are purely stochastic processes. The remaining “reproduction”, “movement”, “mortality” processes become semi-stochastic by implementing discrete probability distributions.

*Collectives:* there are no defined collectives in the model.

*Observation:* output serves to calculate the number of living individuals of a given population at a given time and, consequently, population viability over time.

**LITERATURE CITED**

Anadón, J.D., Giménez, A., Ballestar, R., & Pérez, I. (2009). Evaluation of local ecological knowledge as a method for collecting extensive animal abundance data. *Conservation Biology*, 23, 617-625.

Anadón, J. D., Wiegand, T., & Giménez, A. (2012). Individual‐based movement models reveals sex‐biased effects of landscape fragmentation on animal movement. *Ecosphere*, 3, 1-32.

Caswell, H. (2001). Matrix population models: construction, analysis, and interpretation, Second Edition. ed. Sinauer Associates, Sunderland, Massachusetts, USA.

Cutuli, G., Cannicci, S., Vannini, M., & Fratini, S. (2013). Influence of mating order on courtship displays and stored sperm utilization in Hermann's tortoises (*Testudo hermanni hermanni*). *Behavioral Ecology and Sociobiology*, 67, 273-281.

Díaz-Paniagua, C., Andreu, A. C., & Keller, C. (2006). Effects of temperature on hatching success in field incubating nests of spur-thighed tortoises, *Testudo graeca*. *Herpetological Journal*, 16, 249-257.

Díaz-Paniagua, C., Keller, C., & Andreu, A. C. (1995). Annual variation of activity and daily distances moved in adult Spur-thighed tortoises, *Testudo graeca*, in southwestern Spain. *Herpetologica*, 51, 225-233.

Díaz-Paniagua, C., Keller, C., & Andreu, A. C. (1996). Clutch frequency, egg and clutch characteristics, and nesting activity of spur-thigheed tortoises, *Testudo graeca*, in southwestern Spain. *Canadian Journal of Zoology*, 74, 560-564.

Díaz-Paniagua, C., Keller, C., & Andreu, A. C. (1997). Hatching success, delay of emergence and hatching biometry of *Testudo graeca* in southwestern Spain. *Journal of Zoology*, 243, 543-553.

Díaz-Paniagua, C. Keller, C., & Andreu, A.C. (2001). Long-term demographic fluctuations of the spur-thighed tortoise, *Testudo graeca*, in SW Spain. *Ecography*, 24, 707-721.

Díaz-Paniagua, C., Keller, C., & Andreu, A. C. (2002). Life history and demography of *Testudo greca* in Southwestern Spain. *Chelonii*, 3, 214-222.

Graciá, E., Rodríguez-Caro, R.C., Sanz-Aguilar, A., Anadón, J.D., Botella, F., García-García, A.L., Wiegand, T., Giménez, A. (2020). Assessment of the key evolutionary traits that prevent extinctions in human-altered habitats using a spatially explicit individual-based model. *Ecological Modelling*, 415, 108823.

Jiménez-Franco, M. V., Giménez, A., Rodríguez-Caro, R., Sanz-Aguilar, A., Botella, F., Anadón, J. D., Wiegand, T., & Graciá, E. (2020). Data from: Sperm storage reduces the strength of the mate-finding Allee effect. FigShare Repository. Software. https://doi.org/10.6084/m9.figshare.11498703.v1

Pérez, I., Giménez, A., Anadón, J. D., Martínez, M., & Esteve, M. A. (2002). Patrones de actividad estacional y diaria de la tortuga mora (*Testudo graeca graeca*) en el sureste de la Península Ibércia. *Anales de Biología*, 24, 55-65.

R Core Team. (2016). R: A language and environment for statistical computing. R Foundation for Statistical Computing, Vienna, Austria. URL https://www.R-project.org/.

Roques, S., Díaz-Paniagua, C., & Andreu, A. C. (2004). Microsatellite markers reveal multiple paternity and sperm storage in the Mediterranean spur-thighed tortoise, *Testudo graeca*. *Canadian Journal of Zoology*, 82, 153-159.

Sanz-Aguilar, A., Anadón, J. D., Giménez, A., Ballestar, R., Graciá, E., & Oro, D. (2011). Coexisting with fire: the case of the terrestrial tortoise Testudo graeca in mediterranean shrublands. *Biological Conservation*, 144, 1040-1049.

Stubben, C., & Milligan, B. G. (2007). Estimating and analyzing demographic models using the popbio package in R. *Journal of Statistical Software*, 22, 1-23.
